# Increased pituitary adenylate cyclase-activating peptide genes expression in the prefrontal cortex in schizophrenia in relation to suicide

**DOI:** 10.3389/fnmol.2023.1277958

**Published:** 2023-11-02

**Authors:** Zala Slabe, Rawien A. Balesar, Ronald W. H. Verwer, Gorazd Drevenšek, Dick F. Swaab

**Affiliations:** ^1^Netherlands Institute for Neuroscience, Institute of the Royal Netherlands Academy of Arts and Sciences, Amsterdam, Netherlands; ^2^Institute of Pharmacology and Experimental Toxicology, Faculty of Medicine, University of Ljubljana, Ljubljana, Slovenia

**Keywords:** PACAP, PACAP receptors, prefrontal cortex, schizophrenia, suicide

## Abstract

**Introduction:**

Pituitary adenylate cyclase-activating peptide (PACAP) is a stress-related neuropeptide that is produced in several brain areas. It acts by 3 receptors: PACAP type-1 (PAC1), vasoactive intestinal peptide (VIP) -1 and -2 (VPAC1 and 2). Data on polymorphisms in PACAP and PAC1 indicate a relationship of the PACAP system with schizophrenia (SCZ).

**Methods:**

The prefrontal cortex was chosen to measure PACAP-gene related expression changes, since this is a central structure in the symptoms of schizophrenia (SCZ). We investigated alterations in the expression of the PACAP-related genes by qPCR in the human dorsolateral prefrontal cortex (DLPFC) and anterior cingulate cortex (ACC) of 35 SCZ patients and 34 matched controls in relation to SCZ, suicide, gender and medication.

**Results:**

The ACC revealed an upregulation in PACAP, PAC1, VPAC1 and VPAC2 in SCZ suicide (S) completers compared to controls. An increase in PACAP, VPAC1 and VPAC2 expression was also present in the ACC in SCZ-S compared to SCZ patients who died naturally (SCZ-N). In the DLPFC, an increase in PAC1 was found in SCZ-N patients compared to SCZ-S and controls. Moreover, an increase in all PACAP-related genes was present in SCZ-N male patients compared to SCZ-N females. Concluding, expression changes were found in PACAP-related genes in relation to SCZ, suicide and gender. In particular, there was a higher PACAP-related gene expression in SCZ patients in the ACC in relation to suicide and in DLPFC in relation to SCZ.

**Discussion:**

These findings suggest a potential link between PACAP and the pathophysiology of SCZ and suicide. Further research is needed to understand the functional significance and potential clinical applications of these changes.

## 1. Introduction

Pituitary adenylate cyclase-activating peptide (PACAP) is a stress-related neuropeptide that is presumed to play a key role in disorders which are characterized by enhanced activity of stress systems, including schizophrenia (SCZ). SCZ, is a debilitating psychiatric disorder, that affects about 1% of the global population (Katayama et al., 2009) and is associated with profound disturbances in cognition, perception, and emotional processing (Luvsannyam et al., 2022). Recent evidence has suggested that altered neural networks within the prefrontal cortex (PFC) contribute to cognitive impairments in SCZ (Sakurai et al., 2015).

PACAP is part of the vasoactive intestinal polypeptide (VIP)/secretin/glucagon peptide family and can bind to three different receptors: to a specific G protein-coupled PACAP receptor type I (PAC1) and to two vasoactive intestinal peptide (VIP) receptors, VPAC1 and VPAC2 (Hirabayashi et al., 2018). PACAP and its three receptors are involved in various mood disorders and in post-traumatic stress disorder (Ressler et al., 2011; Lutfy and Shankar, 2019).

PACAP acts as a neuromodulator or neurotransmitter in the nervous system (Grinevich et al., 1997). It stimulates adenylate cyclase as a second messenger resulting in cAMP production (Koide et al., 2014) that influences cAMP signaling pathways (Yan et al., 2016; Yang et al., 2020). Elevated cAMP levels activate protein kinase A, which can phosphorylate ion channels, receptors, and other proteins, increasing intracellular Ca^2+^ concentration, and thus affecting the membrane potential and so the excitability of neurons (Gao et al., 2022). Adenylate cyclase plays this way a crucial role in regulating neuronal excitability, synaptic plasticity (Gilmartin and Ferrara, 2021; May et al., 2021), and in various neuroprotective and neurodegenerative processes (Lee and Seo, 2014).

In addition, a number of observations indicate the existence of a relationship between the PACAP system and SCZ. Variants of the genes encoding components of the PACAP system are associated with susceptibility to SCZ (Hashimoto et al., 2007; Koga et al., 2010; Vacic et al., 2011; Ago et al., 2018; Takeuchi et al., 2020). Previous studies have implicated alterations in the VPAC2 receptor, a key component of the VIP signaling pathway, in the vulnerability of SCZ (Takeuchi et al., 2020; Ago et al., 2021). Moreover, duplication of chromosome 7q36.3, which encodes the VPAC2 receptor, is associated with SCZ (Shen et al., 2013). Overexpression of the VPAC2 receptor has been linked to increased risk for SCZ through its mechanistic role in development of SCZ (Ago et al., 2023), while the same author (Ago et al., 2015) previously found that administration of Ro 25–1,553, a VPAC2 agonist, to postnatal mice leads to its overactivity. Several additional clinical and preclinical studies have shown close relationship between VPAC2 overexpression and SCZ, indicating that VPAC2 might present an important target for drug development (Sakamoto et al., 2021). A VPAC2-selective antagonistic peptide developed by Sakamoto et al. (2018), has been shown to reduce cognitive decline in mouse model of psychiatric disorders (Sakamoto et al., 2021).

The *ADCYAP1* gene that encodes PACAP protein, has been shown to be involved in the various causal pathways in SCZ such as purinergic and glutamatergic signaling as well as oxidative stress pathways. Lang (2006) observed that overexpression of PAC1 in transgenic mice resulted in SCZ-like pathology such as enlarged lateral ventricles and reduced cortical and hippocampal volume. Moreover, immunoprecipitation indicated that interaction between *PACAP,* disrupted-in-Schizophrenia 1 *(DISC1)* and *DISC1-binding zinc-finger protein (DBZ)* may play a role in the pathogenesis of SCZ and other related neuropsychiatric disorders such as schizoaffective and bipolar disorder (Hattori et al., 2012). DISC1 is upregulated by PACAP (Lutfy and Shankar, 2019). Furthermore, it has been found that an ADCYAP1/PACAP-deficit alleviators have beneficial effects in *Adcyap1^−/−^* mouse model for early phase of SCZ (Tiihonen et al., 2021). When studying the distribution of the PACAP-ergic system throughout the human brain, the PFC emerges as a major area of termination of PACAP fibers (Ramikie and Ressler, 2016) and a production site of PACAP (Kirry et al., 2018). Moreover, PFC changes in structure and function correlate with clinical symptoms in SCZ (Collo et al., 2020; Schoonover et al., 2020; Vucurovic et al., 2020). Reduced D1 receptor activation in the PFC is related to negative symptoms of SCZ (Collo et al., 2020). Cognitive deficits in SCZ, are related to alterations in DLPFC neural circuitry (Schoonover et al., 2020). The PFC changes are also a factor in predicting social functioning in SCZ (Vucurovic et al., 2020).

Here we set out to investigate the changes in expression of PACAP and its receptors in SCZ in the dorsolateral prefrontal cortex (DLPFC) and the anterior cingulate cortex (ACC) of the PFC. As untreated SCZ frequently leads to suicidal behavior, and approximately 10% of people with SCZ die by suicide (Sher and Kahn, 2019), special attention was paid to the relationship of the PACAP-related genes and the occurrence of suicide. It is presumed that SCZ patients have a higher probability of suicide because of the impairment of the PFC leading to impulsivity and disturbances in cognitive control (Steiner et al., 2008) and due to impaired judgment during periods of psychosis (Zhang et al., 2020a). Recently we found changes in mRNA expression of PACAP and its receptors in the PFC in relation to suicide. Especially in the ACC, PACAP-mRNA was upregulated in major depressive disorder (MDD) and bipolar disorder (BD) patients who died from suicide compared to MDD patients who died from natural causes (Slabe et al., 2023). In the present study we wanted to see whether a PACAP expression increase occurs in SCZ patients who died of suicide, and is thus independent of the underlying psychiatric disorder too. Therefore, attention is paid to expression changes in relation to suicide.

Notably, gender differences exist in the clinical presentation of SCZ. Men are more vulnerable, have an earlier peak of symptom onset, and suffer from more severe and more negative symptoms than women (Ochoa et al., 2012; Gogos et al., 2019), while females often exhibit milder cognitive symptoms compared to males (Giordano et al., 2021). Sex-specific behavior changes have been observed in VPAC2 overexpressing mice (Ago et al., 2023). Recently Yu et al. (2023), explored gender differences in SCZ by analyzing gene expression in the DLPFC. Females with SCZ exhibited more significant gene expression changes, particularly in genes related to mitochondrial function, ATP metabolism, and neural pathways involving dopamine and GABA (Yu et al., 2023). Oestrogens are involved in PACAP expression, and PACAP is a gender-dependent risk factor for PTSD. A single nucleotide polymorphism (rs2267735) in one of the predicted oestrogen response elements involved in PAC1R-gene regulation is associated with PTSD, but only in women (Ressler et al., 2011; Ramikie and Ressler, 2016). Because of this, our data were also tested for gender differences in the PACAP system in SCZ.

In addition, the effects of antipsychotic use were further considered as a putative confounder in the present study, as it was shown that both typical and atypical neuroleptic drugs influenced the expression of PAC1/VPAC receptors in a glioblastoma cell line (Jóźwiak-Bębenista and Kowalczyk, 2017).

To determine changes in PACAP and its receptor levels, we measured the expression levels of PAC1, VPAC1 and VPAC2 by a reverse transcription-quantitative polymerase chain reaction (RT-qPCR) in the anterior cingulate cortex (ACC) and dorsolateral prefrontal cortex (DLPFC) of the PFC of controls and SCZ patients. Special attention was paid to the influence of suicide, gender and medication.

## 2. Materials and methods

### 2.1. PFC brain samples

Human brain RNA samples (*n* = 69) were obtained from the Stanley Medical Research Institute (SMRI) (Bethesda, MD, USA, Director Dr. Maree Webster). The next of kin provided informed consent for the use of the material. A diagnosis of SCZ was made by experienced senior psychiatrists according to the Diagnostic and Statistical Manual of Mental Disorders (DSM) IV (American Psychiatric Association, 1994). The diagnostic assessments of unaffected control subjects were predicated upon structured interviews conducted by a senior psychiatrist to eliminate Axis I diagnoses. Control subjects who did not exhibit any manifestations of psychiatric or neurological disorders, including the absence of suicidal behaviors, were classified as non-psychiatric controls.

SMRI defined a set of exclusion criteria, comprising:

- The presence of noteworthy structural brain pathology, as confirmed either through post-mortem examination by a qualified neuropathologist or through antemortem imaging.
- A documented history of substantial focal neurological signs preceding death.
- A history of central nervous system disorders known to exert a lasting influence on gene expression.
- A documented intelligence quotient (IQ) below the threshold of 70.
- Evidence of suboptimal RNA quality, as indicated by a RNA integrity value (RIN) falling below the threshold of 7.
- Supplementary exclusion criteria were also implemented for unaffected control subjects, encompassing individuals below the age of 30 (as they were deemed to be within the critical period of maximal risk for schizophrenia) and individuals exhibiting a history of substance abuse within 1 year before decease or notable alcohol-related hepatic alterations.

The specimens were all collected, processed, and stored in a standardized way (The Stanley Medical Research Institute, 2019). The SMRI provided RNA isolated from the grey matter of the post-mortem human anterior cingulate cortex (ACC) and the dorsolateral prefrontal cortex (DLPFC) of “the ARRAY collection,” and provided all demographic information and medical data, including any use of psychotropic medication in their lifetime and a history of drug abuse (Table 1). For all data see Supplementary Tables S1, S2.

**Table 1:** Clinico-pathological information of “Array collection.”

	**Ctr**	**SCZ**	**Value of *p***
Age (year)	45 (31–60)	43 (19–59)	0.622^1^
Gender (M/F)	25/9	26/9	0.943^2^
PMD (hour)	28.5 (9–58)	30 (9–80)	0.631^1^
Brain pH	6.69 (6.00–7.03)	6.50 (5.90–6.93)	0.015^1^
Brain weight (grams)	1,413 (1120–1900)	1,465 (1170–1,630)	0.718^1^
Hemisphere	16 L/18R	17 L/18R	0.9^2^
Age of onset (year)		20 (9–34)	
Duration of illness (year)		24 (1–45)	
Suicide		7	
Psychotic features		35	
RIN (SD)	7 (0.68) DLPFC	7.5 (0.53) DLPFC	0.1^1^ DLPFC
	7 (1.05) ACC	7 (0.67) ACC	0.14^1^ ACC

ACC, anterior cingulate cortex, Ctr, control; DLPFC, dorsolateral prefrontal cortex; F, female; L, left; M, male; PMD, post mortem delay; R, right; SD, standard deviation; SCZ, schizophrenia. ^1^Mann–Whitney test, data showed with median and range. ^2^Chi-square test.

The “ARRAY collection” consists of 35 schizophrenic (SCZ) patients [7 suicide completers (SCZ-S) and 28 patients who died of natural causes (SCZ-N)] and 34 well-matched controls without psychiatric or neurological disease (Table 1). All the analyses were performed blind in terms of the diagnosis of the patients.

### 2.2. Quantitative real-time PCR (RT-qPCR)

Complementary DNA (cDNA) synthesis was prepared according to previous research done by our group (Zhao et al., 2018). For complementary DNA (cDNA) synthesis, an equal quantity of RNA (500 ng in a 10 μL reaction) for each sample was transcribed to cDNA using the QuantiTect Reverse Transcription kit (Qiagen, cat. no. 205313), after which cDNA was stored at −20°C or used immediately (see Zhao et al., 2018). According to the manufacturer, the provided RT Primer Mix contains an optimized mix of oligo-dT and random primers. This formulation enables synthesis of high yields of cDNA template, regardless of the location of the amplified target on the transcript. In this way, the sensitivity of low-abundant genes is enhanced significantly.

### 2.3. RT-qPCR

Primers were designed using the online Realtime PCR tool from Integrated DNA Technologies (IDT). We tried to design, as much as possible, intron spanning primers to reduce the risk of genomic DNA detection. Moreover, traces of genomic DNA were already removed from the RNA samples, by treatment with DNase. The melting temperature and GC percentage of primers was generally in the range of 55–62°C and 40–60%, respectively. We avoided primers with secondary structures like dimers and hairpins. Primer length was 18–25 nt.

The primer sequences (Table 2) were checked by BLAST for cross reactivity, and were found to be specific for the target genes.

**Table 2:** Primer sequences of PACAP and its receptors.

**Name**	**Sequences**
**PACAP receptor genes**	
PAC1 forward	GGA-GCA-GGACAG-CAA-CCA
PAC1 reverse	CCT-CGA-TGA-ACAGCC-AGA-AG
VPAC1 forward	TTG-AGG-ATT-ATG-GGT-GCT-GG
VPAC1 reverse	AGT-TTC-TGA-AGC-ATT-CGG
VPAC2 forward	CGG-CAA-CGA-CCA-GTC-TCA-GT
VPAC2 reverse	GAT-GGG-AAA-CAC-GGC-AAA-C
PACAP forward	CTA-GGG-AAG-AGG-TAT-AAA-CAA-AGG-G
PACAP reverse	ACG-AGC-GAT-GAC-TGT-TGA-G
**Reference genes**	**Sequences**
ACTIN forward	CCC-AGC-CAT-GTA-CGT-TGC-TA
ACTIN reverse	TCA-CCG-GAC-TCC-ATC-ATCG-AT
HPRT1 forward	GGA-CAG-GAC-TGA-ACG-TCT-TGC
HPRT1 reverse	ATA-GCC-CCC-CTT-GAG-CAC-AC
TUBα forward	CTT-TGA-GCC-AGC-CAA-CCA
TUBα reverse	GTA-CAA-CAG-GCA-GCA-AGC-CAT
TUBβ forward	GGG-CCA-AGT-TTT-GGG-AGG-T
TUBβ reverse	CAC-TGT-CCC-CAT-GGT-ATG-TGC
UBC forward	ATT-GGG-TCG-CGG-TTC-TTG
UBC reverse	TGC-CTT-GAC-ATT-CTC-GAT-GGT
GAPDH forward	CAA-ATT-CCA-TGG-CAC-CGT-C
GAPDH reverse	TCT-CGC-TCC-TGG-AAG-ATG-GT

The qPCR experiments were begun by determining the primer efficiencies. This was done by creating a cDNA pool through the collection of 1 μL cDNA from different ACC and DLPFC samples. Subsequently, the pooled cDNA was tested at different dilutions, ranging from 1:1 to 1:64 (in two-fold increments). Of each dilution of pooled cDNA, 1 μL was used in a qPCR reaction, consisting of SYBR Green PCR master mix (Lot. No 1903523, Applied Biosystems, CA, USA) and a mixture of forward and reverse primers (each at a final concentration of 0.15 μM) in a total volume of 10 μL. Each qPCR cycle comprised the following steps: 2 min at 50°C; 10 min at 95°C; 1 min at 60°C; 15 s at 95°C; 1 min at 60°C; and 15 s at 96°C (Applied Biosystems 7,300 RealTime PCR system). The efficiency of the primers was calculated using the inverse logarithm of the dilutions. The slope was calculated, and after that, the efficiency was calculated by efficiency = 1/−slope.

To determine the alterations in the PACAP, PAC1, VPAC1 and VPAC2 and CD38 mRNA expression in the MDD, BD and control patients, each qPCR reaction (10 μL volume, see above) was carried out with cDNA, equivalent to a total RNA input of 5 ng. The data were acquired and processed automatically by the Applied Biosystems 7,300 Real Time PCR System.

As negative controls, non-template (NTC) and non-reverse transcriptase (−RT) controls were included. The NTC consisted of replacing the cDNA template with sterile water, while the RT mix was made by omitting reverse transcriptase during the cDNA synthesis. The −RT control serves as a check for contamination with traces of genomic DNA. The chance of influencing the results by variability in the samples was reduced by using measurements of stable reference genes.

#### 2.3.1. Reference genes

Inherent in PCR analysis are sources of variation due to the input of samples and their processing in the PCR machine. This external variation tends to dominate both the biological variation within groups and the explanatory variation between groups. The external variation can be visualized using a pairs plot (a matrix of scatterplots of pairs of all relevant genes) where all pairs show a positive correlation. To reduce the impact of the external variation reference genes can be used. Useful reference genes have a low variability (also called stability *cf.*) (Vandesompele et al., 2002) and reduce the external variation of the set of target genes. For each individual reference gene we evaluated its stability and how much it contributed to the reduction of the external target gene variation. The use of log-transformed observations facilitates the correction with reference genes and also enables the application of conventional statistical methods. A combination of the best performing reference genes was selected and for each group the residuals of the combined reference genes were subtracted from the target gene observations. Here, we have used the combination of ACTIN, alpha-TUBULIN, beta-TUBULIN, GAPDH, HPRT1 and UBC as set of the reference genes.

### 2.4. Statistical analysis

We used nonparametric statistical methods throughout to avoid distributional assumptions that may not be appropriate for our data. Analysis of confounding factors was performed using GraphPad Prism 9.2. (GraphPad Software, 2021). For the statistical analysis of the qPCR results, S+ software (version 8.2) (TIBCO, 2010) was used. Categorical data were analyzed with the Chi-square test. The Spearman test was used to investigate correlations between confounding factors such as pH, age, brain weight and antipsychotic medication in SCZ patients. Comparisons of 2 groups of interval data were assessed by the Mann–Whitney test. For cases with more than 2 groups we used the Kruskal-Wallis test with multiple comparisons (Conover, 1980). All applied tests were two-sided and we considered value of *p*s <0.05 as significant.

Before processing the gene expression data, the values were 10log-transformed to enable simple reference gene correction and conventional statistical procedures. In situations of multiple testing (for instance when a test was applied to several genes) the Benjamini–Hochberg correction (Benjamini and Hochberg, 1995) was used to correct all value of *p*s and an alpha level of 5% was adopted. For the Mann–Whitney test this procedure is straightforward. However, the Kruskal-Wallis test requires that the global value of *p* is significant before multiple comparisons are allowed (Conover, 1980). Therefore, we first corrected the global value of *p*s with the Benjamini–Hochberg procedure. The genes for which the corrected global value of *p* was <0.05 were selected for subsequent multiple comparisons. For each comparison between groups the value of *p*s of selected genes were pooled and were subsequently corrected with the Benjamini–Hochberg procedure.

### 2.5. ANCOVA

Although confounding factors were matched for among the different groups of subjects it may be possible that within the range of a matched confounding factor values correlations exist that reveal aspects of differences between control subjects and patients not detectable by the Mann–Whitney or the Kruskal–Wallis test. Thus, analysis combining both factor variables and regression variables in a linear model (ANCOVA approach) might provide extra information. We used a generalized least squares analysis (gls) (Pinheiro José and Bates, 2000) for this purpose. In this form of analysis various variance and correlation structures of data can be specified. Such a linear model expects a normal distribution of the errors, which is doubtful with the present data. Therefore, we applied this method in an exploratory manner. After examining a model including all relevant covariates of the disease variable [age, postmortem delay (PMD), brain weight, pH of the corticospinal fluid (PH) and the RNA integrity value (RIN)] we decided to concentrate on PMD, PH and RIN. The question of interest is whether inclusion of these covariates in the gls model has an effect on the parameter estimates of the disease variable.

Generally, in the four main divisions (ACC and DLPFC with their subdivisions AC vs. SCZ and AC vs. SCZ-NS vs. SCZ-S) the inclusion of PMD, PH and RIN did not cause major differences in the parameter estimates of the disease groups (data not shown). This means that the conclusions of the Mann–Whitney tests and the Kruskal–Wallis tests were not challenged. There was one noteworthy exception: the difference of PAC1 gene expression between AC and SCZ-S in DLPFC was significant after inclusion of PH in the gls model, while it was not detected by the Kruskal-Wallis test. However, multiple comparisons of the analysis with PH as covariate also declared the difference between AC and SCZ-S as non-significant. Therefore, we consider the conclusions from the Mann–Whitney and Kruskal-Wallis tests as reliable.

## 3. Results

### 3.1. Gene expression alterations in the ACC and DLPFC in SCZ

Following correction for multiple testing, no significant difference was observed in the gene expression of PACAP-related genes in the ACC or DLPFC in SCZ patients compared to their matched controls (Table 3).

**Table 3:** PACAP-related gene expression in the ACC and DLPFC between SCZ patients and matched controls.

	**Fold change**		**Value of *p***	**BHadj-p**
**ACC**	Ctr vs. SCZ			
PACAP	1.2		0.29	
PAC1	1.23		0.1	
VPAC1	1.07		0.3	
VPAC2	−1.1		0.8	
**DLPFC**				
PACAP	−1.1		0.75	
PAC1	1.22		**0.03**	0.12
VPAC1	1.15		0.55	
VPAC2	−1.13		0.99	

ACC, anterior cingulate cortex; BHadj-p, value of *p* following Benjamini–Hochberg corrected value of *p*; Ctr, control; DLPFC, dorsolateral prefrontal cortex; SCZ, schizophrenia. BHadj-p values are not presented when non-corrected values were > 0.05. The bold value represents a significant difference.

### 3.2. Relation to suicide

**Figure 1:**
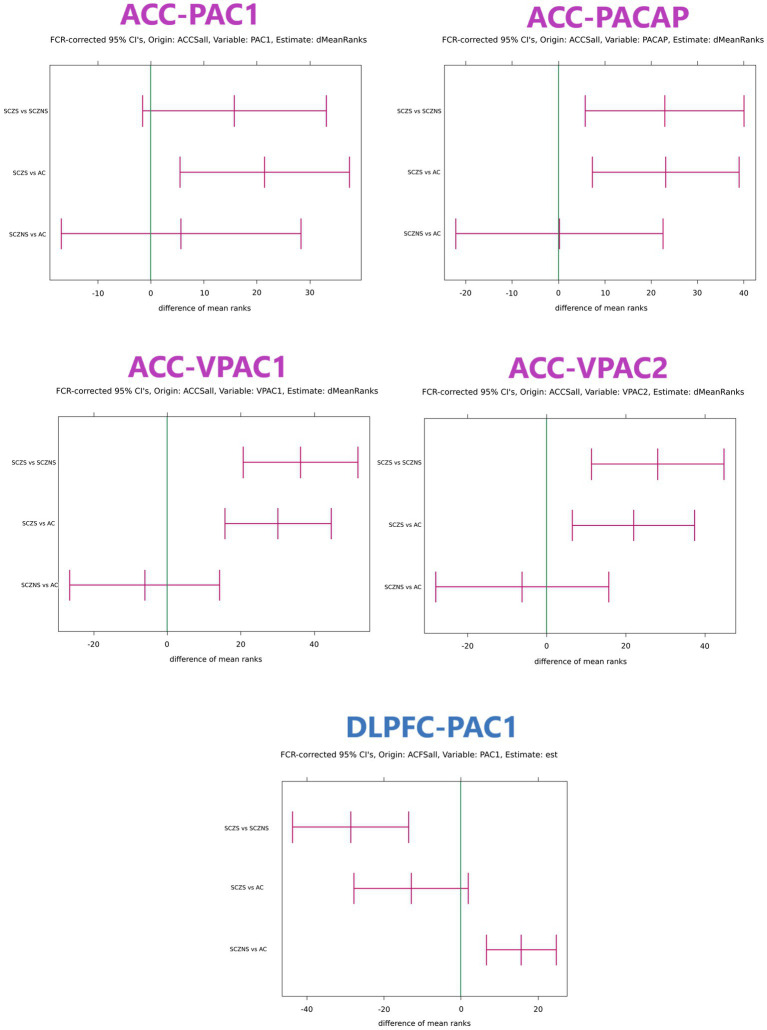
Confidence intervals of differences in target gene expression in ACC and DLPFC with grouping structure AC vs. SCZ-S vs. SCZ-NS. Only comparisons that featured at least 1 significant confidence interval are shown. The confidence intervals were obtained with the Kruskal–Wallis test and the units are expressed in differences between mean ranks. If the mean rank difference for group 1 vs. group 2 is positive, then the observations in group 1 are systematically larger than those in group 2. The reverse is true when the mean rank difference between group 1 and group 2 is negative. The global value of *p*s for all target genes in an area were corrected for multiple testing using the Benjamini–Hochberg False Discovery Rate criterion. For each gene with significant global value of *p*s the confidence intervals for SCZ-NS vs. AC, SCZ-S vs. AC and SCZ-S vs. SCZ-NS per gene were first determined using the multiple comparisons procedure for the Kruskal–Wallis test presented in Conover (1980). In area ACC all target genes (PAC1, PACAP, VPAC1, and VPAC2) had simultaneous significant global value of *p*s and each confidence interval needed to be expanded to comply with 4 simultaneous tests using the Benjamini–Hochberg procedure. This procedure was repeatedly applied for comparisons SCZ-NS vs. AC, for SCZ-S vs. AC and for SCZ-NS vs. SCZ-S. In area DLPFC only target gene PAC1 had a significant global value of *p* and consequently the multiple comparisons for this gene were sufficient without correction for further multiple testing. ACCSall: all reference genes in ACC were used to normalize the target gene expression values. ACFSall: all reference genes in DLPFC were used to normalize the target gene expression values. dMeanRanks or est.: the central values of the estimated difference of the mean ranks. FCR: False Coverage Rate, the confidence interval equivalent of the False Discovery Rate for value of *p*s correction in the Benjamini–Hochberg procedure. AC, control group; SCZ-S, schizophrenic patients who committed suicide; SCZ-NS, schizophrenic patients who did not commit suicide.

There were quite a number of significant differences in the PACAP-related gene expression between suicide completers and SCZ patients who died of natural causes and their matched controls (Table 4; Figure 1).

**Table 4:** PACAP-related gene expression in the ACC and DLPFC in suicide completers, SCZ patients who died of natural causes and their matched controls.

	**Fold change**						**BHadj-p**	**BHadj-p (2 steps)**		
**ACC**	Ctr vs. SCZ-N		Ctr vs. SCZ-S		SCZ-N vs. SCZ-S			Ctr vs. SCZ-N	Ctr vs. SCZ-S	SCZ-N vs. SCZ-S
PACAP	−1.01		2.22	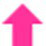	2.24	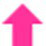	**0.02**	0.96	**0.008**	**0.008**
PAC1	1.31		1.63	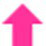	1.24		**0.03**	0.33	**0.009**	0.06
VPAC1	−1.08		1.92	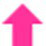	2.09	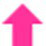	**0.0004**	0.33	**0.0004**	**0.00002**
VPAC2	−1.31		1.70	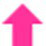	2.24	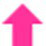	**0.008**	0.33	**0.008**	**0.001**
**DLPFC**										
PACAP	−1.46		1.17		1.71		0.54			
PAC1	1.35	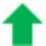	1.25		−1.69	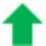	**0.002**	**0.001**	0.09	**0.0003**
VPAC1	1.22		−1.06		−1.29		0.54			
VPAC2	1.15		−1.07		−1.23		0.88			

ACC, anterior cingulate cortex; BHadj-p, value of *p* following Benjamini-Hochberg’s adjustment; Ctr, controls; DLPFC, dorsolateral prefrontal cortex; N, natural death; SCZ, schizophrenia; S, suicide completers; 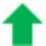 SCZ-N are elevated; 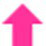 SCZ-S are elevated. BHadj-p values are not presented when non-corrected values were > 0.05. The bold values present significant differences.

#### 3.2.1. ACC

For the three group comparisons, an increase in gene expression in SCZ suicide completers was observed: PACAP (Ctr vs. SCZ-S Fold change = 2.22; BHadj-*p* = 0.008; SCZ-N vs. SCZ-S: Fold change = 2.24; BHadj-p = 0.008), PAC1 (Ctr vs. SCZ-S: Fold change = 1.63; BHadj-*p* = 0.009) and VPAC2 (Ctr vs. SCZ-S Fold change = 2.24; BHadj-p = 0.008; SCZ-N vs. SCZ-S: Fold change = 2.24; BHadj-*p* = 0.001). In addition, a highly significant increase was observed in suicide completers vs. controls: VPAC1 (Ctr vs. SCZ-S: Fold change = 1.92; BHadj-*p* = 0.0004; SCZ-N vs. SCZ-S Fold change = 2.09; BHadj-*p* = 0.00002). No significant alterations were observed between the controls and non-suicidal SCZ patients (Table 4).

#### 3.2.2. DLPFC

For the three group comparisons, an increase in SCZ patients who died of natural causes was observed in PAC1 gene expression (Ctr vs. SCZ-N: Fold change = 1.35; BHadj-*p* = 0.001). In addition, SCZ patients with completed suicide had a lower PAC1 expression than SCZ patients who died of natural causes (SCZ-N vs. SCZ-S: Fold change = −1.69; BHadj-*p* = 0.0003) (Table 4).

### 3.3. Gender differences

Gender differences in PACAP-related gene expression were present in the DLPFC, both in controls and in SCZ patients (see Tables 5, 6).

**Table 5:** Gender difference in PACAP-related gene expression in the ACC and DLPFC in controls and all SCZ patients.

	Fold change		Value of *p*	BHadj-p	Fold change		value of p	BHadj-p
ACC	Ctr F vs. Ctr M				SCZ F vs. SCZ M			
PACAP	−1.16		0.92		1.41		0.24	
PAC1	1.1		0.89		1.2		0.97	
VPAC1	1.42	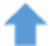	**0.03**	0.12	1.03		0.94	
VPAC2	−1.01		0.65		1.29		1	
DLPFC								
PACAP	−1		0.92		1.7		0.06	
PAC1	1.18		0.45		1.24		0.15	
VPAC1	1.02		0.8		1.8	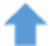	**0.003**	**0.01**
VPAC2	1.7		0.29		3.15		**0.04**	0.08

ACC, anterior cingulate cortex; BHadj-p, p-value following Benjamini-Hochberg’s adjustment; Ctr, control; DLPFC, dorsolateral prefrontal cortex; F, female; M, male; SCZ, schizophrenia; 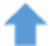 upregulation in males; BHadj-p values are not presented when non-corrected values were > 0.05. The bold values present significant differences.

**Table 6:** Gender difference in PACAP-related gene expression in the ACC and DLPFC in SCZ patients.

	**Fold change**		**value of p**	**BHadj-p**	**Fold change**		**value of p**	**BHadj-p**
**ACC**	SCZ F-N vs. SCZ M-N				SCZ F-S vs. SCZ M-S			
PACAP	1.77		0.06		−1.09		1	
PAC1	−1.12		0.65		1.41		**0.03**	0.14
VPAC1	1.07		0.78		1.52		0.08	
VPAC2	1.46		0.61		1.38		1	
**DLPFC**								
PACAP	4.8	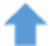	**0.01**	**0.01**	−2.2		0.48	
PAC1	1.72	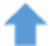	**0.016**	**0.016**	−2.8		**0.03**	0.07
VPAC1	2.8	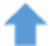	**0.0008**	**0.003**	−2.0		**0.03**	0.07
VPAC2	8.6	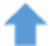	**0.006**	**0.01**	−5.2		0.29	

ACC, anterior cingulate cortex; BHadj-p, value of *p* following Benjamini-Hochberg’s adjustment; Ctr, control; DLPFC, dorsolateral prefrontal cortex; F, female; M, male; N, died of natural causes; SCZ, schizophrenia; S, suicide completers; 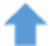 upregulation in males. BHadj-p values are not presented when non-corrected values were > 0.05. The bold values present significant differences.

#### 3.3.1. ACC

No significant gender difference in expression was present in the ACC after correction for multiple testing (Table 5). In addition, no significant gender difference was found between SCZ women who died of natural causes and SCZ men who died of natural causes, for any of the genes (Table 6).

#### 3.3.2. DLPFC

Upregulation of gene expression of VPAC1 (Fold change = 1.8; *p* < 0.003; BHadj-*p* = 0.01) was present in men compared to women in SCZ patients (Table 5).

Non-suicidal SCZ men showed an upregulation in mRNA levels in comparison to non-suicidal SCZ women (Table 6): PACAP (Fold change = 4.8; *p* = 0.01; BHadj-*p* = 0.01); PAC1 (Fold change = 1.72; *p* = 0.016; BHadj-*p* = 0.016); VPAC1 (Fold change = 2.8; *p* < 0.0008; BHadj-*p* < 0.003) and VPAC2 (Fold change = 8.6; *p* < 0.006; BHadj-*p* = 0.01).

### 3.4. Analysis of potentially confounding variables

Correlations between the expression of the PACAP genes and possible confounders were examined. Only the significant results are presented. For all data see Supplementary Tables S3A–C, S4A–C.

In the DLPFC, negative correlations were found in controls between post-mortem delay (PMD) and PAC1 gene expression (rho = −0.54; *p* = 0.01, BHadj-*p* = 0.03), between PMD and VPAC2 gene expression (rho = −0.47; *p* = 0.02, BHadj-*p* = 0.048). The correlations were gender dependent, and only concerned men. The correlations in PMD did not influence our conclusions since they had been matched for.

### 3.5. Correlations with antipsychotics

Antipsychotics were calculated as equivalents of fluphenazine doses (in milligrams) during the lifetime of a patient (see Supplementary Tables S3A–C, S4A–C). No significant correlation was found in the ACC or DLPFC of our data with antipsychotics.

## 4. Discussion

Clear expression changes were found in PACAP-related genes in relation to suicide, gender and medication.

The present study revealed regionally different PACAP-related gene changes in individuals with SCZ who died of suicide. In the ACC, increased expression of PACAP, VPAC1, VPAC2 and PAC1 was observed in donors who died of suicide when compared to SCZ patients who died of natural causes. In contrast, in the DLPFC expression of the PAC1 gene was decreased in suicide completers.

Expression of VPAC1 in the DLPFC was elevated in all SCZ men when compared to SCZ women. An increased expression of all PACAP-related genes was detected in the DLPFC of SCZ men who died of natural causes compared to SCZ women who died of natural causes. Gender differences were present when all SCZ patients were pooled and when those who died of natural causes were examined separately. However, we did not find gender differences in SCZ patients who died of suicide.

### 4.1. PACAP in relation to schizophrenia and suicide

We did not observe significant changes in the PACAP-related genes in the ACC or DLPFC in the overall SCZ group compared to controls. This is in contrast to an animal model study for SCZ, where alterations of PAC1, PACAP and VPAC2 were observed in the frontal cortex (Hashimoto et al., 2007; Jóźwiak-Bębenista and Kowalczyk, 2017).

However, we did find clear changes in PACAP-related genes in relation to suicide. In the ACC of SCZ patients who completed suicide, increased expression of PACAP, PAC1, VPAC1, and VPAC2 was observed, indicating increased sensitivity to PACAP. A duplication of the VPAC2 gene was previously shown to be associated with SCZ (Shen et al., 2013), but no distinction was made in that study between SCZ patients who had suicide ideations or died of suicide or natural causes. In contrast, in the DLPFC only PAC1 was significantly decreased in SCZ subjects who completed suicide, illustrating the selective local changes in the PFC in relation to suicide.

Recently we found increases in mRNA expression of PACAP and its receptors in the ACC in relation to suicide in MDD and BD patients compared to MDD patients who died from natural causes (Slabe et al., 2023). The present study found that a PACAP expression increase occurred in SCZ patients who died of suicide too. This finding implies that the increased PACAP expression in suicide is independent of the underlying psychiatric disorder. Indeed, we found earlier in SCZ patients who died from accomplished suicide compared to SCZ who died from natural causes an upregulation of the purinergic receptor 12 (P2RY12) in the ACC (Zhang et al., 2020b). Since P2RY12 expression was also upregulated in this brain area following suicide in bipolar disorder (BD) and in major depressive disorder (MDD) patients (Zhang et al., 2020b, 2021), the increase in P2RY12. expression appeared to be suicide specific and also independent on the underlying psychiatric disorder.

### 4.2. Relation to GABA-glutamate

Several studies indicate PFC alterations in glutamate and GABA in depression and suicide (Sequeira et al., 2009; Lewis et al., 2020). In the present study, most alterations in PACAP-related genes in SCZ were found in the ACC rather than in the DLPFC. This is in agreement with the glutamate changes observed earlier by us in depression (Zhao et al., 2018). In the ACC, a significantly enhanced expression of genes related to glutamatergic and GABAergic synaptic neurotransmission was found only in MDD donors who committed suicide, whereas in MDD donors who died of natural causes, decreased transcript levels of these genes were found (Zhao et al., 2018) Moreover, in the DLPFC, expression of these genes was decreased in the MDD donors who committed suicide, compared to MDD donors who died from natural causes. Both groups showed increased expression of glutamatergic and GABAergic related genes compared to control subjects (Zhao et al., 2018). VIP and PACAP were shown to interact synergistically with glutamate to increase the ‘throughput’ or ‘strength’ of glutamate-mediated signaling in the cerebral cortex (Magistretti et al., 1998). Such a mechanism may play a role in the ACC in suicide. In patients with recent-onset SCZ and past suicidal ideation or behavior, altered ACC-based circuit function during conflict-monitoring was shown by imaging (Minzenberg et al., 2015). The ACC is a cortical area which has been related to the cognitive evaluation and emotional reaction to pain rather than to the perception of pain itself (Price, 2000). Furthermore, postmortem studies of Schoonover et al. (2020), in the DLPFC shed light on the alterations in both, excitatory pyramidal neurons and inhibitory GABAergic neurons.

Hence, the intense psychological pain prior to the act of suicide may relate to hyperactivity in ACC neurotransmitters. This idea is supported by a coordinate-based meta-analysis of functional MRI studies (van Heeringen et al., 2014), that found greater reactivity of the ACC in suicide attempters when compared to psychiatric controls. Furthermore, a recent near-infrared spectroscopy study of Matsuoka et al. (2020) showed a diminished activation in the right DLPFC in suicidal SCZ patients compared to non-suicidal patients. Future studies are needed to establish whether selectively suppressing ACC activity by targeting GABA and/or glutamatergic synapses locally, e.g., by depth electrodes, could be used as a therapeutic approach towards the prevention of suicide.

### 4.3. Potential effects via glia

In this study, we found an increase of PAC1 expression in the DLPFC in SCZ patients who died of natural causes, compared to controls and suicidal SCZ patients. In an earlier study, we found an astrocytic gene, ALDH1L1, to be elevated in SCZ, especially in the DLPFC of patients who died of natural causes (Zhang et al., 2020a).

Indeed, PACAP receptors are not only present on neurons, but also on astroglia. Astrocytes express both the PACAP-specific receptor PAC1-R and the PACAP/VIP mutual receptors VPAC1-R and VPAC2-R. The presence of these receptors has been described in both resting and reactive brain astrocytes, indicating that some of the actions of PACAP and VIP in the brain may be mediated through astroglia. The involvement of astrocytes in synaptic transmission and synaptogenesis is now clearly established, and the factors released by glial cells that participate in neural communication, such as glutamate, ATP and D-serine, are now coined ‘gliotransmitters’ (Masmoudi-Kouki et al., 2007). The exact relationship between an increase in the PACAP receptor PAC1 that is present on astrocytes and increased expression of astrocytic genes such as ALDH1L1, and their effects, needs further study, since astrocytic dysfunctionality has been previously linked to the development and pathophysiology of schizophrenia (Mei et al., 2018).

### 4.4. Lower brain pH

A significantly lower brain pH was present in the entire SCZ group, which is in line with previous evidence. Lower pH is not considered to be a confounding factor, but rather an endophenotype of this disorder (Dogan et al., 2018; Hagihara et al., 2018). The lower brain pH is thought to be due to increased lactate levels and has also been found *in vivo* by magnetic resonance spectroscopy (MRS). It is not related to medication or clinical variables (Hagihara et al., 2018). A lower brain pH and higher lactate levels were even observed in animal models for psychiatric disorders, including for SCZ. The hypothesis is that decreased oxidative phosphorylation in these disorders will lead to accumulation of lactic acid from glycolysis and subsequent acidification. Antipsychotics may also contribute to the lower brain pH, since they increase lactate levels (Dogan et al., 2018; Hagihara et al., 2018).

### 4.5. Other possible confounders

Well-matched data support our conclusions for decreased PAC1 and VPAC2 gene expression observed in controls having a longer PMD. The confounder correlation analysis did not affect our conclusions, due to the prior well-matched patients.

### 4.6. Antipsychotics

*In vitro* studies showed that antipsychotic drugs may change the expression of PACAP receptors. Following incubation with the typical neuroleptic haloperidol, PAC1 mRNA expression decreased, while all the examined drugs diminished VPAC2 mRNA expression (Jóźwiak-Bębenista and Kowalczyk, 2017). We did not observe any significant correlation between the expression of PACAP-related genes and fluphenazine equivalents during the patients’ lifetime in the ACC.

### 4.7. Gender differences

Women are more susceptible to depression (Albert, 2015) and have a higher prevalence of suicide attempts (Tsirigotis et al., 2011). On the other hand, the prevalence of SCZ is higher and the disease process more serious in men (Karvonen et al., 2007). Therefore, it is not unexpected that we did not find a simple gender difference in the expression of the PACAP-related genes in the different subgroups. Upregulated levels of VPAC1 gene expression was observed in the DLPFC of SCZ men compared to SCZ women. In the DLPFC area all the PACAP-related genes were increased in SCZ men who died of natural causes compared to SCZ women who died of natural causes.

## 5. Limitations

The main finding of the present study is the presence of PACAP-related gene alterations in the PFC of individuals with SCZ who accomplished suicide. Unfortunately, detailed information on suicide attempts and ideations were not available in this forensic material. It is, therefore, possible that some patients who did not die by suicide also had suicide ideations or made suicide attempts. Future studies should reveal to which aspect of suicidality the observed alterations of PACAP-related gene expression are specifically related to.

## 6. Conclusion

PACAP is highly multifunctional neuropeptide involved in many processes (Masmoudi-Kouki et al., 2007; Mei et al., 2018). This study is the first to investigate the changes of PACAP-related gene expression in SCZ patients in the human PFC. The changes in the expression of PACAP-related genes were found to be correlated rather to suicide in SCZ patients than to SCZ *per se*. These changes occurred in particular in the ACC. Furthermore, our investigation unveiled notable alterations in expression of PACAP-related genes, particularly in the context of male gender. Therefore, experimental therapeutic strategies should take the gender of the SCZ patients into consideration. Future studies on the protein level of PACAP and its receptors in SCZ and other neuropsychiatric disorders are now warranted.

## Data availability statement

The datasets for this article are not publicly available due to concerns regarding donors anonymity. Requests to access the datasets should be directed to the corresponding author.

## Ethics statement

The human RNA samples used in this study were acquired from the Stanley Medical Research Institute (Bethesda, MD, USA, Director Dr. Maree Webster). The research was conducted in accordance with the rules of the Stanley Medical Research Institute. Permission was obtained from the next of kin, who provided informed consent for the use of the materials.

## Author contributions

ZS: Data curation, Formal analysis, Investigation, Methodology, Writing – original draft. RB: Supervision, Writing – review & editing. RV: Formal analysis, Software, Visualization, Writing – review & editing. GD: Funding acquisition, Project administration, Writing – review & editing. DFS: Funding acquisition, Project administration, Writing – review & editing, Conceptualization, Resources, Supervision, Validation.
