# Supplementary material for: Increased pituitary adenylate cyclase-activating peptide genes expression in the prefrontal cortex in schizophrenia in relation to suicide
Source: Front Mol Neurosci. 2023 Nov 2;16:1277958. doi: 10.3389/fnmol.2023.1277958 (PMC10652791; doi:10.3389/fnmol.2023.1277958)
Supplement: Supplementary file 1 [file Table_1.DOCX]

**Supplementary material**

**Table S1.** Control patients of Array collection with clinico-pathological information

| Group | Age | Gender | Suicide | Cause of | PMI | Brain  PH | Brain | RIN | |
| --- | --- | --- | --- | --- | --- | --- | --- | --- | --- |
|  |  |  | Status | Death |  |  | Weight | DLPFC | ACC |
| CTR1 | 44 | 2 | 0 | CARDIAC | 28 | 6.59 | 1330 | 8.3 | 7.5 |
| CTR2 | 49 | 1 | 0 | CARDIAC | 46 | 6.5 | 1605 | 8.3 | 7.5 |
| CTR3 | 53 | 1 | 0 | CARDIAC | 9 | 6.4 | 1500 | 9.1 | 8 |
| CTR4 | 37 | 1 | 0 | CARDIAC | 13 | 6.5 | 1600 | 8.3 | 7.8 |
| CTR5 | 51 | 1 | 0 | CARDIAC | 31 | 6.7 | 1400 | 7.3 | 7.6 |
| CTR6 | 53 | 1 | 0 | CARDIAC | 28 | 6 | 1340 | 8.4 | 5.2 |
| CTR7 | 38 | 2 | 0 | CARDIAC | 33 | 6 | 1120 | 9.7 | 7.3 |
| CTR8 | 38 | 2 | 0 | CARDIAC | 28 | 6.7 | 1350 | 8.8 | 7.6 |
| CTR9 | 60 | 1 | 0 | CARDIAC | 47 | 6.8 | 1460 | 8.4 | 6.9 |
| CTR10 | 35 | 1 | 0 | MYOCARDITIS | 52 | 6.7 | 1700 | 8.7 | 8 |
| CTR11 | 34 | 1 | 0 | CARDIAC | 22 | 6.48 | 1480 | 8.3 | 7.7 |
| CTR12 | 47 | 1 | 0 | CARDIAC | 21 | 6.81 | 1550 | 8.7 | 7.5 |
| CTR13 | 45 | 1 | 0 | CARDIAC | 29 | 6.94 | 1405 | 8.5 | 8.6 |
| CTR14 | 34 | 2 | 0 | CARDIAC | 24 | 6.87 | 1255 | 6.6 | 8.8 |
| CTR15 | 42 | 1 | 0 | CARDIAC | 37 | 6.91 | 1340 | 7.8 | 8.4 |
| CTR16 | 44 | 2 | 0 | CARDIAC | 10 | 6.2 | 1305 | 8.4 | 5.6 |
| CTR17 | 45 | 1 | 0 | CARDIAC | 18 | 6.81 | 1585 | 9.5 | 8.8 |
| CTR18 | 49 | 1 | 0 | CARDIAC | 23 | 6.93 | 1390 | 8.3 | 8.7 |
| CTR19 | 32 | 1 | 0 | CARDIAC | 24 | 7.03 | 1415 | 8.3 | 7.5 |
| CTR20 | 55 | 1 | 0 | CARDIAC | 31 | 6.7 | 1515 | 7.9 | 7.5 |
| CTR21 | 49 | 2 | 0 | CARDIAC | 45 | 6.72 | 1435 | 8.3 | 8.8 |
| CTR22 | 33 | 2 | 0 | ASTHMA | 29 | 6.52 | 1360 | 7 | 6.5 |
| CTR23 | 48 | 1 | 0 | CARDIAC | 31 | 6.86 | 1580 | 8 | 7.5 |
| CTR24 | 50 | 1 | 0 | CARDIAC | 49 | 6.75 | 1645 | 7.5 | 8 |
| CTR25 | 32 | 1 | 0 | CARDIAC | 13 | 6.57 | 1410 | 8.3 | 8.9 |
| CTR26 | 47 | 1 | 0 | CARDIAC | 11 | 6.6 | 1495 | 8.6 | 9 |
| CTR27 | 46 | 1 | 0 | CARDIAC | 31 | 6.67 | 1360 | 8.7 | 9.1 |
| CTR28 | 40 | 1 | 0 | CARDIAC | 38 | 6.67 | 1498 | 8.7 | 8.1 |
| CTR29 | 51 | 1 | 0 | CARDIAC | 22 | 6.71 | 1900 | 8.4 | 7.8 |
| CTR30 | 31 | 1 | 0 | PULM EMBOL | 11 | 6.13 | 1335 | 8.6 | 5.2 |
| CTR31 | 48 | 1 | 0 | CARDIAC | 24 | 6.91 | 1321 | 8.4 | 6.4 |
| CTR32 | 39 | 2 | 0 | CARDIAC | 58 | 6.46 | 1260 | 8 | 5.8 |
| CTR33 | 47 | 1 | 0 | CARDIAC | 36 | 6.57 | 1535 | 7.3 | 6.8 |
| CTR34 | 41 | 2 | 0 | CARDIAC | 50 | 6.17 | 1290 | 6.6 | 6.4 |
| Median | 45 |  |  |  | 28.5 | 6.69 | 1413 | 7 | 7 |

*Notes: AC, array collection; CARDIAC, cardiac arrest; PMI, post-mortem interval (hours); PULM EMBOL, Pulmonary embolism; RIN, RNA Integrity Number.*

**Table S2**. Schizophrenic patient of Array collection with clinico-pathological information

| Group | DSM_IV | Age | Gender | Suicide Status | Cause of death | | | PMI | Brain  PH | | Brain Weight | | RIN values | | | | Lifetime antipsychotics | | |  |
| --- | --- | --- | --- | --- | --- | --- | --- | --- | --- | --- | --- | --- | --- | --- | --- | --- | --- | --- | --- | --- |
|  |  |  |  |  | |  |  | | |  | |  | | DLPFC | | ACC | |  | | |
| SCZ1 | 295.9 | 43 | 1 | 0 | PNEUMONIA | | | 26 | 6.42 | | 1620 | | 7.5 | | 7.4 | | 180000 | |  |  |
| SCZ2 | 295.3 | 31 | 1 | 0 | CARDIAC | | | 33 | 6.2 | | 1480 | | 8.5 | | 8.9 | | 35000 | |  |  |
| SCZ3 | 295.3 | 45 | 2 | 1 | SUIC:JUMPED | | | 52 | 6.51 | | 1510 | | 9 | | 8.8 | | 20000 | |  |  |
| SCZ4 | 295.9 | 40 | 1 | 0 | PNEUMONIA | | | 34 | 6.18 | | 1480 | | 8.8 | | 8.6 | | 75000 | |  |  |
| SCZ5 | 295.9 | 51 | 1 | 0 | CARDIAC | | | 43 | 6.63 | | 1390 | | 8.6 | | 7.6 | | 130000 | |  |  |
| SCZ6 | 295.9 | 19 | 1 | 0 | OD | | | 28 | 6.73 | | 1465 | | 9 | | 8.2 | | 2500 | |  |  |
| SCZ7 | 295.9 | 53 | 2 | 0 | CARDIAC | | | 13 | 6.49 | | 1345 | | 8.8 | | 9 | | 15000 | |  |  |
| SCZ8 | 295.9 | 37 | 1 | 0 | CARDIAC | | | 30 | 6.8 | | 1550 | | 9 | | 8.7 | | 20000 | |  |  |
| SCZ9 | 295.1 | 52 | 1 | 0 | CARDIAC | | | 10 | 6.1 | | 1450 | | 8.5 | | 6 | | 100000 | |  |  |
| SCZ10 | 295.9 | 24 | 1 | 1 | SUIC:OD | | | 15 | 6.2 | | 1505 | | 8.4 | | 9 | | 12000 | |  |  |
| SCZ11 | 295.9 | 44 | 1 | 0 | EXHAUST.MANIA/NMS | | | 9 | 5.9 | | 1415 | | 9.4 | | 7.8 | | 350000 | |  |  |
| SCZ12 | 295.3 | 39 | 1 | 0 | MVA | | | 80 | 6.6 | | 1355 | | 8.4 | | 8.8 | | 120000 | |  |  |
| SCZ13 | 295.9 | 33 | 1 | 0 | CARDIAC | | | 29 | 6.5 | | 1470 | | 9.1 | | 8.1 | | 20000 | |  |  |
| SCZ14 | 295.3 | 50 | 1 | 0 | CARDIAC | | | 9 | 6.2 | | 1400 | | 8.4 | | 8.4 | | 34000 | |  |  |
| SCZ15 | 295.9 | 43 | 1 | 0 | CIRRHOSIS | | | 18 | 6.3 | | 1520 | | 8.2 | | 7.7 | | 90000 | |  |  |
| SCZ16 | 295.9 | 32 | 2 | 1 | SUIC:JUMPED | | | 36 | 6.8 | | 1340 | | 8.8 | | 8.6 | | 10000 | |  |  |
| SCZ17 | 295.3 | 35 | 1 | 0 | CARDIAC | | | 47 | 6.4 | | 1370 | | 7.5 | | 7.8 | | 200000 | |  |  |
| SCZ18 | 295.3 | 44 | 1 | 0 | CARDIAC | | | 32 | 6.67 | | 1560 | | 7.7 | | 7.4 | | 20000 | |  |  |
| SCZ19 | 295.9 | 47 | 1 | 0 | ACUTE PANCREATITIS | | | 13 | 6.3 | | 1310 | | 8.3 | | 8.6 | | 300000 | |  |  |
| SCZ20 | 295.9 | 45 | 1 | 0 | CARDIAC | | | 35 | 6.66 | | 1390 | | 7.6 | | 7.4 | | 50 | |  |  |
| SCZ21 | 295.9 | 36 | 2 | 1 | SUIC:HANGING | | | 27 | 6.49 | | 1480 | | 8.4 | | 8.6 | | 600 | |  |  |
| SCZ22 | 295.9 | 54 | 1 | 0 | CARDIAC | | | 38 | 6.17 | | 1400 | | 8.3 | | 7.9 | | 120000 | |  |  |
| SCZ23 | 295.9 | 54 | 2 | 0 | PNEUMONIA | | | 42 | 6.65 | | 1170 | | 8.4 | | 7.8 | | 400000 | |  |  |
| SCZ24 | 295.9 | 44 | 2 | 0 | POSS PULMTHROMB | | | 26 | 6.58 | | 1490 | | 8.7 | | 7.9 | | 50000 | |  |  |
| SCZ25 | 295.9 | 47 | 2 | 0 | OD | | | 30 | 6.47 | | 1430 | | 8.7 | | 7.2 | | 15000 | |  |  |
| SCZ26 | 295.9 | 39 | 1 | 1 | SUIC:HANGING | | | 26 | 6.8 | | 1470 | | 8.2 | | 8 | | 48000 | |  |  |
| SCZ27 | 295.9 | 38 | 1 | 0 | OD | | | 35 | 6.68 | | 1210 | | 8.4 | | 8.7 | | 15000 | |  |  |
| SCZ28 | 295.9 | 41 | 1 | 0 | CARDIAC | | | 54 | 6.18 | | 1629 | | 7.9 | | 7.4 | | 115000 | |  |  |
| SCZ29 | 295.9 | 43 | 1 | 1 | SUIC:HANGING | | | 65 | 6.67 | | 1490 | | 9.6 | | 7.8 | | 70000 | |  |  |
| SCZ30 | 295.9 | 42 | 1 | 1 | SUIC:JUMPED | | | 26 | 6.19 | | 1410 | | 8.3 | | 7.4 | | 10000 | |  |  |
| SCZ31 | 295.3 | 47 | 2 | 0 | CARDIAC | | | 35 | 6.5 | | 1575 | | 9 | | 7.8 | | 90000 | |  |  |
| SCZ32 | 295.3 | 42 | 1 | 0 | CARDIAC | | | 19 | 6.48 | | 1310 | | 9.3 | | 7.9 | | 18000 | |  |  |
| SCZ33 | 295.9 | 46 | 1 | 0 | PNEUMONIA | | | 30 | 6.72 | | 1630 | | 8.8 | | 8.2 | | 200000 | |  |  |
| SCZ34 | 295.9 | 59 | 2 | 0 | CARDIAC | | | 38 | 6.93 | | 1515 | | 7.6 | | 7.3 | | 30000 | |  |  |
| SCZ35 | 295.9 | 52 | 1 | 0 | PNEUMONIA | | | 16 | 6.52 | | 1340 | | 7.7 | | 6.7 | | 60000 | |  |  |
| Median |  | 43 |  |  |  | | | 30 | 6.50 | | 1465 | | 7.5 | | 7 | |  | |  |  |

*Notes: AC, array collection; CARDIAC, cardiac arrest; DSM_IV, diagnosis; EXHAUST MANIA, exhaustive mania; Lifetime Antipsychotics, in milligrams (mg); MVA, Mosaic variegated aneuploidy; NMS, Neuroleptic malignant syndrome; OD=overdose; PMI, post-mortem interval (hours); 1, male; 2, female;* POSS PULMTHROMB, thrombolysis in pulmonary embolism; *RIN, RNA Integrity Numbe; SUIC, suicide; Suicide status, 1-suicide completed, 0-died of natural causes.*

**Table S3. A**

| **SCZ ACC** |  |  |  |  |  | **SCZ-N ACC** | |  |  |  |
| --- | --- | --- | --- | --- | --- | --- | --- | --- | --- | --- |
| **Confounding factor** | Gene | rho | p.value | BHadj.p |  | **Confounding factor** | Gene | rho | p.value | BHadj.p |
| **age** | VPAC1 | -0.29 | 0.09 | 0.24 |  | **age** | PACAP | -0.24 | 0.21 | 0.83 |
| **age** | PACAP | -0.27 | 0.12 | 0.24 |  | **age** | VPAC1 | -0.16 | 0.42 | 0.83 |
| **age** | VPAC2 | -0.17 | 0.33 | 0.43 |  | **age** | VPAC2 | -0.09 | 0.64 | 0.86 |
| **age** | PAC1 | -0.09 | 0.6 | 0.6 |  | **age** | PAC1 | -0.03 | 0.88 | 0.88 |
|  |  |  |  |  |  |  | **var2** |  |  |  |
| **PMI** | VPAC2 | 0.21 | 0.22 | 0.74 |  | **PMI** | VPAC2 | 0.23 | 0.23 | 0.9 |
| **PMI** | PACAP | -0.11 | 0.53 | 0.74 |  | **PMI** | VPAC1 | 0.08 | 0.67 | 0.91 |
| **PMI** | PAC1 | -0.1 | 0.55 | 0.74 |  | **PMI** | PACAP | -0.05 | 0.81 | 0.91 |
| **PMI** | VPAC1 | 0.02 | 0.88 | 0.88 |  | **PMI** | PAC1 | 0.02 | 0.91 | 0.91 |
|  |  |  |  |  |  |  | **var2** |  |  |  |
| **PH** | VPAC2 | 0.22 | 0.21 | 0.54 |  | **PH** | PAC1 | -0.23 | 0.24 | 0.67 |
| **PH** | PAC1 | -0.19 | 0.27 | 0.54 |  | **PH** | VPAC2 | 0.14 | 0.45 | 0.67 |
| **PH** | PACAP | 0.06 | 0.72 | 0.76 |  | **PH** | VPAC1 | -0.11 | 0.55 | 0.67 |
| **PH** | VPAC1 | -0.05 | 0.76 | 0.76 |  | **PH** | PACAP | 0.08 | 0.67 | 0.67 |
|  |  |  |  |  |  |  | **var2** |  |  |  |
| **BW** | PAC1 | 0.34 | 0.05 | 0.2 |  | **BW** | PAC1 | 0.35 | 0.07 | 0.28 |
| **BW** | VPAC1 | 0.15 | 0.37 | 0.53 |  | **BW** | VPAC2 | 0.15 | 0.43 | 0.65 |
| **BW** | PACAP | 0.13 | 0.45 | 0.53 |  | **BW** | VPAC1 | 0.12 | 0.53 | 0.65 |
| **BW** | VPAC2 | 0.11 | 0.53 | 0.53 |  | **BW** | PACAP | 0.09 | 0.65 | 0.65 |
|  |  |  |  |  |  |  | **var2** |  |  |  |
| **Lifetime Antipsychotics** | PAC1 | 0.18 | 0.29 | 0.9 |  | **Lifetime Antipsychotics** | PAC1 | 0.31 | 0.11 | 0.44 |
| **Lifetime Antipsychotics** | VPAC1 | -0.06 | 0.72 | 0.9 |  | **Lifetime Antipsychotics** | VPAC1 | 0.17 | 0.38 | 0.59 |
| **Lifetime Antipsychotics** | PACAP | -0.05 | 0.75 | 0.9 |  | **Lifetime Antipsychotics** | VPAC2 | 0.15 | 0.44 | 0.59 |
| **Lifetime Antipsychotics** | VPAC2 | -0.02 | 0.9 | 0.9 |  | **Lifetime Antipsychotics** | PACAP | 0.1 | 0.6 | 0.6 |
|  |  |  |  |  |  |  |  |  |  |  |
| **SCZ-S ACC** | |  |  |  |  | **Controls ACC** | |  |  |  |
| **Confounding factor** | Gene | rho | p.value | BHadj.p |  | **Confounding factor** | Gene | rho | p.value | BHadj.p |
| **age** | PAC1 | -0.21 | 0.63 | 0.97 |  | **age** | VPAC1 | 0.1 | 0.56 | 0.78 |
| **age** | VPAC2 | 0.14 | 0.76 | 0.97 |  | **age** | VPAC2 | 0.09 | 0.62 | 0.78 |
| **age** | PACAP | 0.07 | 0.9 | 0.97 |  | **age** | PACAP | 0.07 | 0.69 | 0.78 |
| **age** | VPAC1 | -0.04 | 0.97 | 0.97 |  | **age** | PAC1 | 0.05 | 0.78 | 0.78 |
|  | **var2** |  |  |  |  |  |  |  |  |  |
| **PMI** | PAC1 | -0.67 | 0.11 | 0.27 |  | **PMI** | PAC1 | -0.2 | 0.26 | 0.65 |
| **PMI** | VPAC1 | -0.63 | 0.13 | 0.27 |  | **PMI** | VPAC1 | 0.1 | 0.57 | 0.65 |
| **PMI** | PACAP | -0.38 | 0.38 | 0.5 |  | **PMI** | VPAC2 | 0.1 | 0.57 | 0.65 |
| **PMI** | VPAC2 | -0.05 | 0.93 | 0.93 |  | **PMI** | PACAP | 0.08 | 0.65 | 0.65 |
|  | **var2** |  |  |  |  |  |  |  |  |  |
| **PH** | VPAC2 | 0.4 | 0.35 | 0.88 |  | **PH** | VPAC1 | 0.15 | 0.39 | 0.79 |
| **PH** | VPAC1 | -0.29 | 0.51 | 0.88 |  | **PH** | VPAC2 | 0.15 | 0.4 | 0.79 |
| **PH** | PAC1 | -0.2 | 0.66 | 0.88 |  | **PH** | PACAP | 0.08 | 0.63 | 0.85 |
| **PH** | PACAP | -0.02 | 1 | 1 |  | **PH** | PAC1 | 0.02 | 0.92 | 0.92 |
|  | **var2** |  |  |  |  |  |  |  |  |  |
| **BW** | VPAC2 | -0.79 | 0.06 | 0.24 |  | **BW** | VPAC1 | 0.13 | 0.44 | 0.86 |
| **BW** | PAC1 | -0.39 | 0.36 | 0.72 |  | **BW** | PACAP | 0.08 | 0.63 | 0.86 |
| **BW** | VPAC1 | -0.18 | 0.69 | 0.93 |  | **BW** | PAC1 | -0.06 | 0.73 | 0.86 |
| **BW** | PACAP | 0.04 | 0.97 | 0.97 |  | **BW** | VPAC2 | 0.03 | 0.86 | 0.86 |
|  | **var2** |  |  |  |  |  |  |  |  |  |
| **Lifetime Antipsychotics** | PACAP | -0.18 | 0.69 | 1 |  |  |  |  |  |  |
| **Lifetime Antipsychotics** | PAC1 | -0.11 | 0.83 | 1 |  |  |  |  |  |  |
| **Lifetime Antipsychotics** | VPAC2 | 0.09 | 0.86 | 1 |  |  |  |  |  |  |
| **Lifetime Antipsychotics** | VPAC1 | -0.02 | 1 | 1 |  |  |  |  |  |  |

*Notes: ACC, anterior cingulate cortex; BW, brain weight; DLPFC, dorsolateral prefrontal cortex; Lifetime Antipsychotics, fluphenazine doses (in milligrams); N, schizophrenic patients who died naturally; pH, cerebrospinal fluid pH; PMI, post-mortem interval; S, suicide completers; SCZ, schizophrenia.*

| **Table S3. B**  **SCZ Male ACC** | |  |  |  |  | **SCZ Female ACC** | |  |  |  |
| --- | --- | --- | --- | --- | --- | --- | --- | --- | --- | --- |
| **Confounding factor** | Gene | rho | p.value | BHadj.p |  | **Confounding factor** | Gene | rho | p.value | BHadj.p |
| **age** | VPAC1 | -0.21 | 0.29 | 0.72 |  | **age** | VPAC1 | -0.55 | 0.12 | 0.30 |
| **age** | PACAP | -0.15 | 0.44 | 0.72 |  | **age** | PACAP | -0.52 | 0.15 | 0.30 |
| **age** | VPAC2 | -0.12 | 0.54 | 0.72 |  | **age** | VPAC2 | -0.20 | 0.59 | 0.70 |
| **age** | PAC1 | 0.02 | 0.92 | 0.92 |  | **age** | PAC1 | -0.14 | 0.70 | 0.70 |
|  | **var2** |  |  |  |  | **var1** | **var2** | **rho** | **p.value** | **BHadj.p** |
| **PMI** | VPAC2 | 0.34 | 0.09 | 0.36 |  | **PMI** | PAC1 | -0.62 | 0.09 | 0.34 |
| **PMI** | VPAC1 | 0.09 | 0.66 | 0.82 |  | **PMI** | PACAP | -0.20 | 0.59 | 0.94 |
| **PMI** | PACAP | -0.05 | 0.81 | 0.82 |  | **PMI** | VPAC1 | -0.13 | 0.72 | 0.94 |
| **PMI** | PAC1 | -0.05 | 0.82 | 0.82 |  | **PMI** | VPAC2 | -0.03 | 0.94 | 0.94 |
|  | **var2** |  |  |  |  | **var1** | **var2** | **rho** | **p.value** | **BHadj.p** |
| **PH** | VPAC2 | 0.27 | 0.18 | 0.51 |  | **PH** | VPAC1 | -0.22 | 0.55 | 0.83 |
| **PH** | PACAP | 0.23 | 0.25 | 0.51 |  | **PH** | PACAP | -0.17 | 0.65 | 0.83 |
| **PH** | PAC1 | -0.12 | 0.57 | 0.75 |  | **PH** | PAC1 | -0.12 | 0.76 | 0.83 |
| **PH** | VPAC1 | 0.04 | 0.83 | 0.83 |  | **PH** | VPAC2 | 0.08 | 0.83 | 0.83 |
|  | **var2** |  |  |  |  |  | **var2** |  |  |  |
| **BW** | PAC1 | 0.39 | 0.05 | 0.22 |  | **BW** | VPAC2 | -0.42 | 0.25 | 0.98 |
| **BW** | VPAC2 | 0.27 | 0.17 | 0.34 |  | **BW** | PACAP | -0.10 | 0.80 | 0.98 |
| **BW** | VPAC1 | 0.16 | 0.43 | 0.44 |  | **BW** | PAC1 | 0.07 | 0.87 | 0.98 |
| **BW** | PACAP | 0.16 | 0.44 | 0.44 |  | **BW** | VPAC1 | 0.02 | 0.98 | 0.98 |
|  | **var2** |  |  |  |  | **var1** | **var2** | **rho** | **p.value** | **BHadj.p** |
| **Lifetime Antipsychotics** | PAC1 | 0.19 | 0.35 | 0.98 |  | **Lifetime Antipsychotics** | VPAC2 | -0.50 | 0.16 | 0.35 |
| **Lifetime Antipsychotics** | VPAC2 | 0.07 | 0.73 | 0.98 |  | **Lifetime Antipsychotics** | VPAC1 | -0.49 | 0.18 | 0.35 |
| **Lifetime Antipsychotics** | VPAC1 | -0.02 | 0.92 | 0.98 |  | **Lifetime Antipsychotics** | PACAP | -0.40 | 0.27 | 0.35 |
| **Lifetime Antipsychotics** | PACAP | 0.004 | 0.98 | 0.98 |  | **Lifetime Antipsychotics** | PAC1 | 0.02 | 0.98 | 0.98 |
|  |  |  |  |  |  |  |  |  |  |  |
|  |  |  |  |  |  |  |  |  |  |  |
| **SCZ-S Male ACC** | |  |  |  |  | **SCZ-S Female ACC** | | |  |  |
| **Confounding factor** | Gene | rho | p.value | BHadj.p |  | **Confounding factor** | Gene | rho | p.value | BHadj.p |
| **age** | PACAP | -0.4 | 0.60 | 0.80 |  | **age** | VPAC1 | -0.55 | 0.12 | 0.3 |
| **age** | PAC1 | -0.4 | 0.60 | 0.80 |  | **age** | PACAP | -0.52 | 0.15 | 0.3 |
| **age** | VPAC1 | -0.4 | 0.60 | 0.80 |  | **age** | VPAC2 | -0.2 | 0.59 | 0.7 |
| **age** | VPAC2 | 0.2 | 0.86 | 0.86 |  | **age** | PAC1 | -0.14 | 0.7 | 0.7 |
|  | **var2** |  |  |  |  |  | **var2** |  |  |  |
| **PMI** | PACAP | -0.32 | 0.71 | 0.71 |  | **PMI** | PAC1 | -0.62 | 0.09 | 0.34 |
| **PMI** | PAC1 | -0.32 | 0.71 | 0.71 |  | **PMI** | PACAP | -0.2 | 0.59 | 0.94 |
| **PMI** | VPAC1 | -0.32 | 0.71 | 0.71 |  | **PMI** | VPAC1 | -0.13 | 0.72 | 0.94 |
| **PMI** | VPAC2 | 0.32 | 0.71 | 0.71 |  | **PMI** | VPAC2 | -0.03 | 0.94 | 0.94 |
|  | **var2** |  |  |  |  |  | **var2** |  |  |  |
| **PH** | VPAC2 | 0.4 | 0.60 | 0.86 |  | **PH** | VPAC1 | -0.22 | 0.55 | 0.83 |
| **PH** | PACAP | 0.2 | 0.86 | 0.86 |  | **PH** | PACAP | -0.17 | 0.65 | 0.83 |
| **PH** | PAC1 | 0.2 | 0.86 | 0.86 |  | **PH** | PAC1 | -0.12 | 0.76 | 0.83 |
| **PH** | VPAC1 | 0.2 | 0.86 | 0.86 |  | **PH** | VPAC2 | 0.08 | 0.83 | 0.83 |
|  | **var2** |  |  |  |  |  | **var2** |  |  |  |
| **BW** | VPAC2 | -0.8 | 0.23 | 0.39 |  | **BW** | VPAC2 | -0.5 | 0.16 | 0.35 |
| **BW** | PACAP | -0.6 | 0.39 | 0.39 |  | **BW** | VPAC1 | -0.49 | 0.18 | 0.35 |
| **BW** | PAC1 | -0.6 | 0.39 | 0.39 |  | **BW** | PACAP | -0.4 | 0.27 | 0.35 |
| **BW** | VPAC1 | -0.6 | 0.39 | 0.39 |  | **BW** | PAC1 | 0.02 | 0.98 | 0.98 |
|  | **var2** |  |  |  |  |  | **var2** |  |  |  |
| **Lifetime Antipsychotics** | PACAP | -0.4 | 0.60 | 0.80 |  | **Lifetime Antipsychotics** | VPAC2 | -0.5 | 0.16 | 0.35 |
| **Lifetime Antipsychotics** | PAC1 | -0.4 | 0.60 | 0.80 |  | **Lifetime Antipsychotics** | VPAC1 | -0.49 | 0.18 | 0.35 |
| **Lifetime Antipsychotics** | VPAC1 | -0.4 | 0.60 | 0.80 |  | **Lifetime Antipsychotics** | PACAP | -0.4 | 0.27 | 0.35 |
| **Lifetime Antipsychotics** | VPAC2 | 0 | 1 | 1 |  | **Lifetime Antipsychotics** | PAC1 | 0.02 | 0.98 | 0.98 |

*Notes: ACC, anterior cingulate cortex; BW, brain weight; DLPFC, dorsolateral prefrontal cortex; Lifetime Antipsychotics, fluphenazine doses (in milligrams); N, schizophrenic patients who died naturally; pH, cerebrospinal fluid pH; PMI, post-mortem interval; S, suicide completers; SCZ, schizophrenia.*

**Table S3. C**

| **SCZ-N Male ACC** | | |  |  |  | **SCZ-N Female ACC** | | |  |  |
| --- | --- | --- | --- | --- | --- | --- | --- | --- | --- | --- |
| **Confounding factor** | Gene | rho | p.value | BHadj.p |  | **Confounding factor** | Gene | rho | p.value | BHadj.p |
| **age** | VPAC2 | -0.15 | 0.48 | 0.67 |  | **age** | VPAC2 | 0.7 | 0.2 | 0.6 |
| **age** | PACAP | -0.11 | 0.63 | 0.67 |  | **age** | PAC1 | -0.5 | 0.3 | 0.7 |
| **age** | VPAC1 | -0.1 | 0.64 | 0.67 |  | **age** | VPAC1 | -0.2 | 0.7 | 0.8 |
| **age** | PAC1 | 0.09 | 0.67 | 0.67 |  | **age** | PACAP | -0.1 | 0.8 | 0.8 |
|  | **var2** |  |  |  |  |  | **var2** |  |  |  |
| **PMI** | VPAC2 | 0.37 | 0.09 | 0.36 |  | **PMI** | PAC1 | -0.5 | 0.3 | 0.4 |
| **PMI** | VPAC1 | 0.19 | 0.38 | 0.75 |  | **PMI** | PACAP | -0.5 | 0.3 | 0.4 |
| **PMI** | PACAP | 0.07 | 0.75 | 0.8 |  | **PMI** | VPAC1 | -0.5 | 0.3 | 0.4 |
| **PMI** | PAC1 | 0.06 | 0.8 | 0.8 |  | **PMI** | VPAC2 | 0.1 | 0.9 | 0.9 |
|  | **var2** |  |  |  |  |  | **var2** |  |  |  |
| **PH** | PACAP | 0.22 | 0.31 | 0.47 |  | **PH** | VPAC2 | 0.31 | 0.52 | 1 |
| **PH** | VPAC2 | 0.22 | 0.32 | 0.47 |  | **PH** | VPAC1 | -0.14 | 0.8 | 1 |
| **PH** | PAC1 | -0.2 | 0.35 | 0.47 |  | **PH** | PACAP | 0.03 | 1 | 1 |
| **PH** | VPAC1 | -0.03 | 0.88 | 0.88 |  | **PH** | PAC1 | 0.03 | 1 | 1 |
|  | **var2** |  |  |  |  |  | **var2** |  |  |  |
| **BW** | PAC1 | 0.4 | 0.07 | 0.27 |  | **BW** | VPAC2 | -0.43 | 0.37 | 0.89 |
| **BW** | VPAC2 | 0.29 | 0.18 | 0.35 |  | **BW** | PACAP | -0.37 | 0.44 | 0.89 |
| **BW** | PACAP | 0.15 | 0.49 | 0.56 |  | **BW** | PAC1 | 0.03 | 1 | 1 |
| **BW** | VPAC1 | 0.13 | 0.56 | 0.56 |  | **BW** | VPAC1 | 0.03 | 1 | 1 |
|  | **var2** |  |  |  |  |  | **var2** |  |  |  |
| **Lifetime Antipsychotics** | PAC1 | 0.31 | 0.16 | 0.59 |  | **Lifetime Antipsychotics** | PAC1 | 0.2 | 0.7 | 0.85 |
| **Lifetime Antipsychotics** | VPAC2 | 0.15 | 0.49 | 0.59 |  | **Lifetime Antipsychotics** | PACAP | 0.12 | 0.85 | 0.85 |
| **Lifetime Antipsychotics** | VPAC1 | 0.15 | 0.51 | 0.59 |  | **Lifetime Antipsychotics** | VPAC1 | 0.12 | 0.85 | 0.85 |
| **Lifetime Antipsychotics** | PACAP | 0.12 | 0.59 | 0.59 |  | **Lifetime Antipsychotics** | VPAC2 | 0.12 | 0.85 | 0.85 |
|  |  |  |  |  |  |  |  |  |  |  |
|  |  |  |  |  |  |  |  |  |  |  |
| **Control Males ACC** | | |  |  |  | **Control Females ACC** | | |  |  |
| **Confounding factor** | **Gene** | **rho** | **p.value** | **BHadj.p** |  | **Confounding factor** | **Gene** | **rho** | **p.value** | **BHadj.p** |
| **age** | PAC1 | 0.13 | 0.52 | 0.68 |  | **age** | VPAC1 | 0.1 | 0.56 | 0.78 |
| **age** | PACAP | 0.12 | 0.57 | 0.68 |  | **age** | VPAC2 | 0.09 | 0.62 | 0.78 |
| **age** | VPAC1 | 0.11 | 0.61 | 0.68 |  | **age** | PACAP | 0.07 | 0.69 | 0.78 |
| **age** | VPAC2 | 0.08 | 0.68 | 0.68 |  | **age** | PAC1 | 0.05 | 0.78 | 0.78 |
|  | **var2** |  |  |  |  |  | **var2** | **rho** | **p.value** | **BHadj.p** |
| **PMI** | PACAP | 0.29 | 0.16 | 0.27 |  | **PMI** | PAC1 | -0.2 | 0.26 | 0.65 |
| **PMI** | VPAC2 | 0.27 | 0.19 | 0.27 |  | **PMI** | VPAC1 | 0.1 | 0.57 | 0.65 |
| **PMI** | VPAC1 | 0.26 | 0.2 | 0.27 |  | **PMI** | VPAC2 | 0.1 | 0.57 | 0.65 |
| **PMI** | PAC1 | -0.07 | 0.75 | 0.75 |  | **PMI** | PACAP | 0.08 | 0.65 | 0.65 |
|  | **var2** |  |  |  |  |  | **var2** | **rho** | **p.value** | **BHadj.p** |
| **PH** | PACAP | -0.11 | 0.61 | 0.98 |  | **PH** | VPAC1 | 0.15 | 0.39 | 0.79 |
| **PH** | PAC1 | -0.06 | 0.78 | 0.98 |  | **PH** | VPAC2 | 0.15 | 0.4 | 0.79 |
| **PH** | VPAC1 | -0.02 | 0.93 | 0.98 |  | **PH** | PACAP | 0.08 | 0.63 | 0.85 |
| **PH** | VPAC2 | 0.004 | 0.98 | 0.98 |  | **PH** | PAC1 | 0.02 | 0.92 | 0.92 |
|  | **var2** |  |  |  |  |  | **var2** |  |  |  |
| **BW** | PACAP | 0.16 | 0.45 | 0.87 |  | **BW** | VPAC1 | 0.13 | 0.44 | 0.86 |
| **BW** | VPAC1 | 0.12 | 0.55 | 0.87 |  | **BW** | PACAP | 0.08 | 0.63 | 0.86 |
| **BW** | VPAC2 | 0.05 | 0.8 | 0.87 |  | **BW** | PAC1 | -0.06 | 0.73 | 0.86 |
| **BW** | PAC1 | -0.03 | 0.87 | 0.87 |  | **BW** | VPAC2 | 0.03 | 0.86 | 0.86 |

*Notes: ACC, anterior cingulate cortex; BW, brain weight; DLPFC, dorsolateral prefrontal cortex; Lifetime Antipsychotics, fluphenazine doses (in milligrams); N, schizophrenic patients who died naturally; pH, cerebrospinal fluid pH; PMI, post-mortem interval; S, suicide completers; SCZ, schizophrenia.*

**Table S4. A**

| **Controls DLPFC** | |  |  |  |  | **SCZ DLPFC** | |  |  |  |
| --- | --- | --- | --- | --- | --- | --- | --- | --- | --- | --- |
| **Confounding factor** | **Gene** | **rho** | **p.value** | **BHadj.p** |  | **Confounding factor** | **Gene** | **rho** | **p.value** | **BHadj.p** |
| **age** | PAC1 | -0.26 | 0.14 | 0.53 |  | **age** | PAC1 | 0.28 | 0.16 | 0.56 |
| **age** | PACAP | -0.16 | 0.36 | 0.53 |  | **age** | VPAC2 | 0.22 | 0.28 | 0.56 |
| **age** | VPAC2 | -0.15 | 0.39 | 0.53 |  | **age** | VPAC1 | 0.08 | 0.69 | 0.76 |
| **age** | VPAC1 | -0.10 | 0.56 | 0.56 |  | **age** | PACAP | 0.06 | 0.76 | 0.76 |
|  |  |  |  |  |  |  |  |  |  |  |
|  |  |  |  |  |  |  |  |  |  |  |
| **PMI** | PAC1 | -0.53 | 0.00 | 0.01 |  | **PMI** | PACAP | 0.25 | 0.21 | 0.82 |
| **PMI** | VPAC2 | -0.45 | 0.01 | 0.02 |  | **PMI** | PAC1 | -0.07 | 0.71 | 0.90 |
| **PMI** | VPAC1 | -0.15 | 0.38 | 0.51 |  | **PMI** | VPAC1 | 0.05 | 0.82 | 0.90 |
| **PMI** | PACAP | -0.03 | 0.87 | 0.87 |  | **PMI** | VPAC2 | 0.03 | 0.90 | 0.90 |
|  |  |  |  |  |  |  |  |  |  |  |
|  |  |  |  |  |  |  |  |  |  |  |
| **PH** | VPAC1 | 0.22 | 0.21 | 0.51 |  | **PH** | PACAP | 0.26 | 0.19 | 0.65 |
| **PH** | PACAP | 0.19 | 0.27 | 0.51 |  | **PH** | PAC1 | -0.12 | 0.56 | 0.65 |
| **PH** | VPAC2 | 0.15 | 0.38 | 0.51 |  | **PH** | VPAC1 | 0.11 | 0.58 | 0.65 |
| **PH** | PAC1 | -0.03 | 0.87 | 0.87 |  | **PH** | VPAC2 | -0.09 | 0.65 | 0.65 |
|  |  |  |  |  |  |  |  |  |  |  |
|  |  |  |  |  |  |  |  |  |  |  |
| **BW** | PACAP | 0.23 | 0.19 | 0.77 |  | **BW** | PACAP | -0.33 | 0.10 | 0.34 |
| **BW** | PAC1 | -0.11 | 0.54 | 0.82 |  | **BW** | VPAC2 | -0.28 | 0.18 | 0.34 |
| **BW** | VPAC1 | 0.09 | 0.61 | 0.82 |  | **BW** | VPAC1 | -0.23 | 0.26 | 0.34 |
| **BW** | VPAC2 | -0.01 | 0.96 | 0.96 |  | **BW** | PAC1 | -0.13 | 0.53 | 0.53 |
|  |  |  |  |  |  |  |  |  |  |  |
|  |  |  |  |  |  |  |  |  |  |  |
|  |  |  |  |  |  | **Lifetime Antipsychotics** | VPAC2 | 0.23 | 0.27 | 0.57 |
|  |  |  |  |  |  | **Lifetime Antipsychotics** | PACAP | -0.20 | 0.33 | 0.57 |
|  |  |  |  |  |  | **Lifetime Antipsychotics** | VPAC1 | -0.16 | 0.43 | 0.57 |
|  |  |  |  |  |  | **Lifetime Antipsychotics** | PAC1 | 0.03 | 0.90 | 0.90 |
|  |  |  |  |  |  |  |  |  |  |  |
|  | | |  |  |  |  | |  |  |  |
| **SCZ Females DLPFC** | | |  |  |  | **SCZ Males DLPFC** | |  |  |  |
| **Confounding factor** | **Gene** | **rho** | **p.value** | **BHadj.p** |  | **Confouding**  **factor** | **Gene** | **rho** | **p.value** | **BHadj.p** |
| **age** | VPAC2 | -0.68 | 0.06 | 0.17 |  | **age** | PAC1 | 0.28 | 0.16 | 0.56 |
| **age** | PACAP | -0.62 | 0.08 | 0.17 |  | **age** | VPAC2 | 0.22 | 0.28 | 0.56 |
| **age** | PAC1 | -0.41 | 0.26 | 0.34 |  | **age** | VPAC1 | 0.08 | 0.69 | 0.76 |
| **age** | VPAC1 | -0.33 | 0.36 | 0.36 |  | **age** | PACAP | 0.06 | 0.76 | 0.76 |
|  |  |  |  |  |  |  |  |  |  |  |
|  |  |  |  |  |  |  |  |  |  |  |
| **PMI** | VPAC2 | 0.30 | 0.41 | 0.62 |  | **PMI** | PACAP | 0.25 | 0.21 | 0.82 |
| **PMI** | PACAP | 0.25 | 0.49 | 0.62 |  | **PMI** | PAC1 | -0.07 | 0.71 | 0.90 |
| **PMI** | PAC1 | 0.23 | 0.52 | 0.62 |  | **PMI** | VPAC1 | 0.05 | 0.82 | 0.90 |
| **PMI** | VPAC1 | 0.18 | 0.62 | 0.62 |  | **PMI** | VPAC2 | 0.03 | 0.90 | 0.90 |
|  |  |  |  |  |  |  |  |  |  |  |
|  |  |  |  |  |  |  |  |  |  |  |
| **PH** | VPAC2 | 0.48 | 0.18 | 0.64 |  | **PH** | PACAP | 0.26 | 0.19 | 0.65 |
| **PH** | PACAP | 0.35 | 0.33 | 0.64 |  | **PH** | PAC1 | -0.12 | 0.56 | 0.65 |
| **PH** | PAC1 | 0.26 | 0.48 | 0.64 |  | **PH** | VPAC1 | 0.11 | 0.58 | 0.65 |
| **PH** | VPAC1 | 0.10 | 0.79 | 0.79 |  | **PH** | VPAC2 | -0.09 | 0.65 | 0.65 |
|  |  |  |  |  |  |  |  |  |  |  |
|  |  |  |  |  |  |  |  |  |  |  |
| **BW** | PAC1 | 0.52 | 0.15 | 0.49 |  | **BW** | PACAP | -0.33 | 0.10 | 0.34 |
| **BW** | VPAC1 | 0.38 | 0.29 | 0.49 |  | **BW** | VPAC2 | -0.28 | 0.18 | 0.34 |
| **BW** | PACAP | 0.25 | 0.49 | 0.49 |  | **BW** | VPAC1 | -0.23 | 0.26 | 0.34 |
| **BW** | VPAC2 | 0.25 | 0.49 | 0.49 |  | **BW** | PAC1 | -0.13 | 0.53 | 0.53 |
|  |  |  |  |  |  |  |  |  |  |  |
|  |  |  |  |  |  |  |  |  |  |  |
| **Lifetime Antipsychotics** | PACAP | -0.29 | 0.42 | 0.89 |  | **Lifetime Antipsychotics** | VPAC2 | 0.23 | 0.27 | 0.57 |
| **Lifetime Antipsychotics** | VPAC1 | -0.21 | 0.57 | 0.89 |  | **Lifetime Antipsychotics** | PACAP | -0.20 | 0.33 | 0.57 |
| **Lifetime Antipsychotics** | PAC1 | -0.08 | 0.85 | 0.89 |  | **Lifetime Antipsychotics** | VPAC1 | -0.16 | 0.43 | 0.57 |
| **Lifetime Antipsychotics** | VPAC2 | -0.06 | 0.89 | 0.89 |  | **Lifetime Antipsychotics** | PAC1 | 0.03 | 0.90 | 0.90 |

*Notes: ACC, anterior cingulate cortex; BW, brain weight; DLPFC, dorsolateral prefrontal cortex; Lifetime Antipsychotics, fluphenazine doses (in milligrams); N, schizophrenic patients who died naturally; pH, cerebrospinal fluid pH; PMI, post-mortem interval; S, suicide completers; SCZ, schizophrenia.*

**Table S4. B**

| **SCZ-N DLPFC** | |  |  |  |  | **SCZ-S DLPFC** | |  |  |  |
| --- | --- | --- | --- | --- | --- | --- | --- | --- | --- | --- |
| **Confounding factor** | **Gene** | **rho** | **p.value** | **BHadj.p** |  | **Confounding factor** | **Gene** | **rho** | **p.value** | **BHadj.p** |
| **age** | PACAP | -0.21 | 0.27 | 0.60 |  | **age** | PAC1 | -0.39 | 0.36 | 0.97 |
| **age** | VPAC1 | -0.19 | 0.33 | 0.60 |  | **age** | VPAC1 | -0.25 | 0.57 | 0.97 |
| **age** | VPAC2 | -0.15 | 0.45 | 0.60 |  | **age** | PACAP | -0.04 | 0.97 | 0.97 |
| **age** | PAC1 | -0.04 | 0.83 | 0.83 |  | **age** | VPAC2 | -0.04 | 0.97 | 0.97 |
|  |  |  |  |  |  |  |  |  |  |  |
|  |  |  |  |  |  |  |  |  |  |  |
| **PMI** | PACAP | 0.20 | 0.29 | 0.90 |  | **PMI** | PACAP | 0.41 | 0.33 | 1.00 |
| **PMI** | VPAC1 | 0.04 | 0.85 | 0.90 |  | **PMI** | VPAC1 | 0.04 | 0.96 | 1.00 |
| **PMI** | VPAC2 | 0.04 | 0.86 | 0.90 |  | **PMI** | PAC1 | -0.02 | 1.00 | 1.00 |
| **PMI** | PAC1 | -0.02 | 0.90 | 0.90 |  | **PMI** | VPAC2 | -0.02 | 1.00 | 1.00 |
|  |  |  |  |  |  |  |  |  |  |  |
|  |  |  |  |  |  |  |  |  |  |  |
| **PH** | PACAP | 0.24 | 0.22 | 0.72 |  | **PH** | VPAC2 | 0.40 | 0.35 | 0.83 |
| **PH** | VPAC2 | -0.14 | 0.49 | 0.72 |  | **PH** | VPAC1 | -0.14 | 0.76 | 0.83 |
| **PH** | VPAC1 | 0.12 | 0.54 | 0.72 |  | **PH** | PACAP | 0.13 | 0.79 | 0.83 |
| **PH** | PAC1 | -0.07 | 0.72 | 0.72 |  | **PH** | PAC1 | -0.11 | 0.83 | 0.83 |
|  |  |  |  |  |  |  |  |  |  |  |
|  |  |  |  |  |  |  |  |  |  |  |
| **BW** | VPAC2 | -0.11 | 0.58 | 0.79 |  | **BW** | PACAP | -0.64 | 0.13 | 0.50 |
| **BW** | PAC1 | 0.10 | 0.60 | 0.79 |  | **BW** | VPAC2 | -0.32 | 0.46 | 0.84 |
| **BW** | PACAP | -0.08 | 0.68 | 0.79 |  | **BW** | VPAC1 | -0.21 | 0.63 | 0.84 |
| **BW** | VPAC1 | -0.05 | 0.79 | 0.79 |  | **BW** | PAC1 | -0.07 | 0.90 | 0.90 |
|  |  |  |  |  |  |  |  |  |  |  |
|  |  |  |  |  |  |  |  |  |  |  |
| **Lifetime Antipsychotics** | VPAC2 | 0.24 | 0.23 | 0.93 |  | **Lifetime Antipsychotics** | VPAC1 | -0.86 | 0.04 | 0.10 |
| **Lifetime Antipsychotics** | VPAC1 | -0.09 | 0.64 | 0.93 |  | **Lifetime Antipsychotics** | PAC1 | -0.81 | 0.05 | 0.10 |
| **Lifetime Antipsychotics** | PACAP | -0.06 | 0.77 | 0.93 |  | **Lifetime Antipsychotics** | PACAP | -0.61 | 0.15 | 0.19 |
| **Lifetime Antipsychotics** | PAC1 | -0.02 | 0.93 | 0.93 |  | **Lifetime Antipsychotics** | VPAC2 | -0.13 | 0.79 | 0.79 |
|  |  |  |  |  |  |  |  |  |  |  |
|  | | |  |  |  |  | |  |  |  |
| **SCZ-S Females DLPFC** | | |  |  |  | **SCZ-S Males DLPFC** | |  |  |  |
| **Confounding factor** | **Gene** | **rho** | **p.value** | **BHadj.p** |  | **Confounding factor** | **Gene** | **rho** | **p.value** | **BHadj.p** |
| **age** | PACAP | -1.00 | 0.29 | 0.72 |  | **age** | PACAP | 0.80 | 0.23 | 0.45 |
| **age** | PAC1 | -0.50 | 0.72 | 0.72 |  | **age** | PAC1 | -0.80 | 0.23 | 0.45 |
| **age** | VPAC1 | -0.50 | 0.72 | 0.72 |  | **age** | VPAC1 | -0.40 | 0.60 | 0.60 |
| **age** | VPAC2 | -0.50 | 0.72 | 0.72 |  | **age** | VPAC2 | -0.40 | 0.60 | 0.60 |
|  |  |  |  |  |  |  |  |  |  |  |
|  |  |  |  |  |  |  |  |  |  |  |
| **PMI** | PAC1 | -1.00 | 0.29 | 0.58 |  | **PMI** | PAC1 | -0.95 | 0.14 | 0.48 |
| **PMI** | VPAC1 | -1.00 | 0.29 | 0.58 |  | **PMI** | PACAP | 0.63 | 0.36 | 0.48 |
| **PMI** | PACAP | -0.50 | 0.72 | 0.72 |  | **PMI** | VPAC1 | -0.63 | 0.36 | 0.48 |
| **PMI** | VPAC2 | 0.50 | 0.72 | 0.72 |  | **PMI** | VPAC2 | -0.32 | 0.71 | 0.71 |
|  |  |  |  |  |  |  |  |  |  |  |
|  |  |  |  |  |  |  |  |  |  |  |
| **PH** | VPAC2 | 1.00 | 0.29 | 0.72 |  | **PH** | VPAC1 | -0.80 | 0.23 | 0.77 |
| **PH** | PACAP | 0.50 | 0.72 | 0.72 |  | **PH** | PAC1 | -0.60 | 0.39 | 0.77 |
| **PH** | PAC1 | -0.50 | 0.72 | 0.72 |  | **PH** | PACAP | -0.40 | 0.60 | 0.80 |
| **PH** | VPAC1 | -0.50 | 0.72 | 0.72 |  | **PH** | VPAC2 | 0.20 | 0.86 | 0.86 |
|  |  |  |  |  |  |  |  |  |  |  |
|  |  |  |  |  |  |  |  |  |  |  |
| **BW** | PACAP | -1.00 | 0.29 | 0.72 |  | **BW** | PACAP | -0.80 | 0.23 | 0.77 |
| **BW** | PAC1 | -0.50 | 0.72 | 0.72 |  | **BW** | VPAC2 | -0.60 | 0.39 | 0.77 |
| **BW** | VPAC1 | -0.50 | 0.72 | 0.72 |  | **BW** | VPAC1 | -0.40 | 0.60 | 0.80 |
| **BW** | VPAC2 | -0.50 | 0.72 | 0.72 |  | **BW** | PAC1 | 0.20 | 0.86 | 0.86 |
|  |  |  |  |  |  |  |  |  |  |  |
|  |  |  |  |  |  |  |  |  |  |  |
| **Lifetime Antipsychotics** | PAC1 | -1.00 | 0.29 | 0.58 |  | **Lifetime Antipsychotics** | VPAC1 | -1.00 | 0.12 | 0.45 |
| **Lifetime Antipsychotics** | VPAC1 | -1.00 | 0.29 | 0.58 |  | **Lifetime Antipsychotics** | PAC1 | -0.80 | 0.23 | 0.45 |
| **Lifetime Antipsychotics** | PACAP | -0.50 | 0.72 | 0.72 |  | **Lifetime Antipsychotics** | VPAC2 | -0.40 | 0.60 | 0.80 |
| **Lifetime Antipsychotics** | VPAC2 | 0.50 | 0.72 | 0.72 |  | **Lifetime Antipsychotics** | PACAP | -0.20 | 0.86 | 0.86 |

*Notes: ACC, anterior cingulate cortex; BW, brain weight; DLPFC, dorsolateral prefrontal cortex; Lifetime Antipsychotics, fluphenazine doses (in milligrams); N, schizophrenic patients who died naturally; pH, cerebrospinal fluid pH; PMI, post-mortem interval; S, suicide completers; SCZ, schizophrenia.*

**Table S4. C**

| **SCZ-N Females DLPFC** | | |  |  |  | **SCZ-N Males DLPFC** | |  |  |  |
| --- | --- | --- | --- | --- | --- | --- | --- | --- | --- | --- |
| **Confounding factor** | **Gene** | **rho** | **p.value** | **BHadj.p** |  | **Confounding factor** | **Gene** | **rho** | **p.value** | **BHadj.p** |
| **age** | VPAC1 | 0.41 | 0.40 | 1.00 |  | **age** | PAC1 | 0.21 | 0.34 | 0.84 |
| **age** | VPAC2 | -0.32 | 0.52 | 1.00 |  | **age** | VPAC2 | 0.19 | 0.42 | 0.84 |
| **age** | PACAP | 0.06 | 0.95 | 1.00 |  | **age** | VPAC1 | 0.05 | 0.84 | 0.95 |
| **age** | PAC1 | -0.03 | 1.00 | 1.00 |  | **age** | PACAP | 0.02 | 0.95 | 0.95 |
|  |  |  |  |  |  |  |  |  |  |  |
|  |  |  |  |  |  |  |  |  |  |  |
| **PMI** | PAC1 | 0.49 | 0.31 | 0.81 |  | **PMI** | PACAP | 0.20 | 0.35 | 0.89 |
| **PMI** | PACAP | 0.37 | 0.44 | 0.81 |  | **PMI** | VPAC2 | 0.10 | 0.66 | 0.89 |
| **PMI** | VPAC1 | 0.26 | 0.61 | 0.81 |  | **PMI** | VPAC1 | 0.05 | 0.82 | 0.89 |
| **PMI** | VPAC2 | 0.09 | 0.90 | 0.90 |  | **PMI** | PAC1 | -0.03 | 0.89 | 0.89 |
|  |  |  |  |  |  |  |  |  |  |  |
|  |  |  |  |  |  |  |  |  |  |  |
| **PH** | PACAP | 0.60 | 0.20 | 0.33 |  | **PH** | PACAP | 0.37 | 0.09 | 0.36 |
| **PH** | PAC1 | 0.54 | 0.25 | 0.33 |  | **PH** | VPAC1 | 0.23 | 0.28 | 0.57 |
| **PH** | VPAC2 | 0.54 | 0.25 | 0.33 |  | **PH** | VPAC2 | -0.03 | 0.88 | 0.93 |
| **PH** | VPAC1 | 0.37 | 0.44 | 0.44 |  | **PH** | PAC1 | -0.02 | 0.93 | 0.93 |
|  |  |  |  |  |  |  |  |  |  |  |
|  |  |  |  |  |  |  |  |  |  |  |
| **BW** | PAC1 | 0.83 | 0.07 | 0.19 |  | **BW** | PACAP | -0.32 | 0.15 | 0.53 |
| **BW** | PACAP | 0.77 | 0.10 | 0.19 |  | **BW** | VPAC2 | -0.26 | 0.26 | 0.53 |
| **BW** | VPAC2 | 0.60 | 0.20 | 0.25 |  | **BW** | VPAC1 | -0.19 | 0.40 | 0.53 |
| **BW** | VPAC1 | 0.54 | 0.25 | 0.25 |  | **BW** | PAC1 | -0.10 | 0.65 | 0.65 |
|  |  |  |  |  |  |  |  |  |  |  |
|  |  |  |  |  |  |  |  |  |  |  |
| **Lifetime Antipsychotics** | PACAP | 0.58 | 0.22 | 0.29 |  | **Lifetime Antipsychotics** | VPAC1 | -0.21 | 0.33 | 0.58 |
| **Lifetime Antipsychotics** | PAC1 | 0.58 | 0.22 | 0.29 |  | **Lifetime Antipsychotics** | PACAP | -0.20 | 0.35 | 0.58 |
| **Lifetime Antipsychotics** | VPAC2 | 0.58 | 0.22 | 0.29 |  | **Lifetime Antipsychotics** | VPAC2 | 0.18 | 0.44 | 0.58 |
| **Lifetime Antipsychotics** | VPAC1 | 0.29 | 0.56 | 0.56 |  | **Lifetime Antipsychotics** | PAC1 | -0.11 | 0.60 | 0.60 |
|  |  |  |  |  |  |  |  |  |  |  |
|  | | |  |  |  |  | |  |  |  |
| **Control Females DLPFC** | | |  |  |  | **Control Males DLPFC** | |  |  |  |
| **Confounding factor** | **Gene** | **rho** | **p.value** | **BHadj.p** |  | **Confounding factor** | **Gene** | **rho** | **p.value** | **BHadj.p** |
| **age** | VPAC1 | 0.58 | 0.11 | 0.42 |  | **age** | PAC1 | -0.36 | 0.08 | 0.23 |
| **age** | PAC1 | 0.34 | 0.35 | 0.58 |  | **age** | VPAC2 | -0.33 | 0.12 | 0.23 |
| **age** | VPAC2 | 0.29 | 0.43 | 0.58 |  | **age** | PACAP | -0.25 | 0.21 | 0.26 |
| **age** | PACAP | -0.19 | 0.60 | 0.60 |  | **age** | VPAC1 | -0.23 | 0.26 | 0.26 |
|  |  |  |  |  |  |  |  |  |  |  |
|  |  |  |  |  |  |  |  |  |  |  |
| **PMI** | PAC1 | -0.65 | 0.07 | 0.27 |  | **PMI** | PAC1 | -0.55 | 0.008 | 0.03 |
| **PMI** | VPAC1 | -0.37 | 0.31 | 0.56 |  | **PMI** | VPAC2 | -0.47 | 0.024 | 0.048 |
| **PMI** | VPAC2 | -0.29 | 0.42 | 0.56 |  | **PMI** | VPAC1 | -0.17 | 0.41 | 0.55 |
| **PMI** | PACAP | -0.08 | 0.83 | 0.83 |  | **PMI** | PACAP | -0.03 | 0.9 | 0.9 |
|  |  |  |  |  |  |  |  |  |  |  |
|  |  |  |  |  |  |  |  |  |  |  |
| **PH** | VPAC1 | 0.50 | 0.16 | 0.39 |  | **PH** | VPAC2 | 0.22 | 0.3 | 0.74 |
| **PH** | PACAP | 0.47 | 0.19 | 0.39 |  | **PH** | VPAC1 | 0.18 | 0.37 | 0.74 |
| **PH** | VPAC2 | -0.10 | 0.80 | 0.94 |  | **PH** | PACAP | 0.11 | 0.59 | 0.79 |
| **PH** | PAC1 | -0.03 | 0.94 | 0.94 |  | **PH** | PAC1 | 0 | 1 | 1 |
|  |  |  |  |  |  |  |  |  |  |  |
|  |  |  |  |  |  |  |  |  |  |  |
| **BW** | PACAP | -0.35 | 0.33 | 0.80 |  | **BW** | PACAP | 0.24 | 0.24 | 0.64 |
| **BW** | VPAC2 | -0.30 | 0.41 | 0.80 |  | **BW** | VPAC1 | 0.2 | 0.32 | 0.64 |
| **BW** | PAC1 | -0.15 | 0.69 | 0.80 |  | **BW** | VPAC2 | -0.08 | 0.69 | 0.75 |
| **BW** | VPAC1 | 0.10 | 0.80 | 0.80 |  | **BW** | PAC1 | -0.06 | 0.75 | 0.75 |

*Notes: ACC, anterior cingulate cortex; BW, brain weight; DLPFC, dorsolateral prefrontal cortex; Lifetime Antipsychotics, fluphenazine doses (in milligrams); N, schizophrenic patients who died naturally; pH, cerebrospinal fluid pH; PMI, post-mortem interval; S, suicide completers; SCZ, schizophrenia.*
